# A Self-Tuning Minimal-Rule Fuzzy Logic Controller for High-Performance Induction Motor Drives

**DOI:** 10.3390/s26154789

**Published:** 2026-07-28

**Authors:** Fuad Alhaj Omar, Nihat Pamuk, Talha Enes Gümüş, Selçuk Emiroğlu

**Affiliations:** 1 Department of Electric and Energy, Zonguldak Bülent Ecevit University, Zonguldak 67100, Türkiye; 2 Department of Electrical and Electronics Engineering, Zonguldak Bülent Ecevit University, Zonguldak 67100, Türkiye; 3 Department of Electrical and Electronics Engineering, Sakarya University, Sakarya 54187, Türkiye; tgumus@sakarya.edu.tr (T.E.G.); selcukemiroglu@sakarya.edu.tr (S.E.)

**Keywords:** minimal-rule controller, fuzzy logic control, induction motor drives, particle swarm optimization, Lyapunov stability, discrete-time control, adaptive control

## Abstract

This paper presents a self-tuning minimal-rule fuzzy logic controller for high-performance induction motor drives operating under field-oriented control. Unlike conventional full-rule fuzzy controllers and reduced-rule designs with fixed post-design scaling, the proposed method combines a fixed nine-rule Mamdani inference structure with a bounded online output gain adaptation mechanism. The nominal gain and adaptation sensitivity are determined offline using Particle Swarm Optimization, thereby retaining operating-condition responsiveness without requiring online optimization, rule reconstruction, or membership-function retuning. The closed-loop behavior is analyzed using a discrete-time Lyapunov framework derived from the induction motor mechanical dynamics under bounded disturbances. The controller is evaluated through fixed-step simulations incorporating measurement noise, 12-bit signal quantization, and a one-sample computational delay. Comparative results against a conventional PI controller and a classical 49-rule fuzzy controller show that the proposed scheme achieves a rise time of 0.15 s, a settling time of 0.26 s, a post-transient mean absolute tracking error of 4 RPM, negligible overshoot, and a torque ripple of approximately 0.44 Nm. Relative to the classical 49-rule FLC, the proposed design reduces the maximum number of fuzzy-rule evaluations per control update from 49 to 9, corresponding to an 81.6% reduction in structural fuzzy-inference complexity. The results indicate a favorable simulation-level trade-off between dynamic performance, disturbance rejection, and structural algorithmic simplicity. Generated-code SIL, Hardware-in-the-Loop testing, target-processor timing measurements, and experimental implementation remain necessary to establish practical embedded feasibility.

## 1. Introduction

Induction motors (IMs) underpin a vast share of modern motion and process control thanks to their simple construction, robustness, competitive cost, and acceptable efficiency. They are the workhorse of industry, responsible for more than 70% of electrical energy consumption in industrial settings, powering fans, compressors, pumps, conveyors, and increasingly electric-traction applications [1]. Despite this ubiquity, IMs exhibit strongly nonlinear, coupled electromechanical dynamics that complicate precise speed regulation, particularly under variable operating points and load disturbances [2].

Scalar strategies such as V/f control are attractive for their simplicity but typically deliver modest dynamics and limited accuracy [3]. Vector control, field-oriented control (FOC), addresses these shortcomings by decoupling flux and torque, thereby improving transient and steady-state behavior; however, FOC performance is sensitive to parameter drift (e.g., rotor resistance, stator inductance) and often hinges on careful tuning and reliable parameter estimation [4].

To contend with nonlinearities and uncertainties without relying on exact models, intelligent control methods have gained traction. Among them, fuzzy logic control (FLC) is notable for embedding heuristic expertise in linguistic rules and membership functions, allowing effective decision-making when precise analytical descriptions are impractical [5,6]. In motor drives, FLCs have repeatedly shown resilience to disturbances and parameter variations, offering smooth torque and robust tracking [7,8]. Empirical evidence includes improvements over classical PI regulators in dynamic response [9] and enhanced performance under varying loads when optimization techniques, such as Particle Swarm Optimization (PSO), tune the fuzzy parameters [10]. These findings reinforce the promise of fuzzy strategies for reliable IM operation.

A persistent drawback of conventional FLC, however, is computational burden. A typical two-input design with seven membership functions per input yields a 49-rule base, overhead that strains embedded platforms and limits the achievable sampling rates [11,12]. To alleviate this, prior work explored rule-base reduction and context-aware pruning. Examples include context-sensitive optimization [13] and simplified fuzzy structures validated in simulation [6]. Yet many proposals retain static architecture and provide limited adaptability in real time.

To address these gaps, the present study introduces a self-tuning fuzzy logic controller with a deliberately minimal rule base for high-performance IM drives. The controller uses just nine rules (3 × 3), cutting complexity while a built-in self-tuning mechanism adjusts the output gain online as a function of speed-error behavior. The combination aims to preserve the favorable transients and steady-state precision associated with richer fuzzy designs, while maintaining a substantially reduced fuzzy-inference structure suitable for future embedded evaluation. The proposed scheme is developed and assessed in MATLAB/Simulink R2024a using a detailed model of a 1.5 kW squirrel-cage IM. In addition to the reference simulations, a fixed-step discrete-time assessment is conducted under modeled measurement noise, signal quantization, and one-sample delay. We benchmark it against a conventional PI controller and a standard 49-rule FLC. Test scenarios include step changes in speed reference and abrupt load perturbations. Performance is quantified via rise time, settling time, overshoot, torque ripple, and robustness to load variations, enabling a comparison under identical simulation conditions, but not under identical offline optimization procedures.

The novelty of the proposed approach lies in the coordinated treatment of rule-based complexity, online adaptability, stability, and digital implementation rather than in any individual component alone. First, the controller preserves a fixed nine-rule inference structure and confines online adaptation to a bounded scalar output gain, avoiding real-time rule modification, membership-function optimization, or iterative search. Second, PSO is used exclusively offline to determine the nominal gain and adaptation sensitivity, thereby transferring the optimization burden outside the control cycle while retaining online responsiveness to speed-error variations. Third, the resulting controller is analyzed in discrete time through Lyapunov-based boundedness analysis and an additional fixed-step simulation incorporating one-sample delay, measurement noise, and signal quantization. This integrated design distinguishes the proposed scheme from reduced-rule controllers that remain statically scaled and from adaptive fuzzy controllers whose improved flexibility may require larger rule bases or more intensive online computation.

The remainder of this paper is organized as follows. Section 2 presents the literature review of control strategies for induction motor drives, with emphasis on fuzzy and adaptive approaches. Section 3 describes the proposed modeling framework and controller design, including the minimal-rule fuzzy structure, adaptive-gain formulation, Lyapunov-based stability analysis, and PSO-based parameter tuning. Section 4 details the simulation setup and reproducibility conditions adopted to ensure fair and consistent benchmarking. Section 5 reports and discusses the comparative results under reference tracking, torque ripple evaluation, and load disturbance scenarios. Section 6 summarizes the key findings and performance–complexity trade-offs. Section 7 examines the simulated closed-loop behavior under fixed-step sampling, measurement noise, signal quantization, and one-sample delay. Finally, Section 8 concludes the paper with the main takeaways and implications for future embedded motor-drive evaluation.

## 2. Literature Review

Over the last several decades, induction motor (IM) control has drawn sustained interest, driven by the need for high-performance drives across industrial automation, transportation, and energy applications. Classical regulators, Proportional–Integral (PI) and field-oriented control (FOC), are widely adopted, yet their robustness degrades when dynamics become nonlinear, time-varying, or uncertain [4,14,15]. This gap motivated the turn toward intelligent controllers, with fuzzy logic emerging in the 1980s as a practical alternative.

Fuzzy logic controllers (FLCs) rely on linguistic rules and membership functions to encode expert knowledge rather than detailed physics-based models. The seminal work of Mamdani and Assilian [16] established the foundations of modern fuzzy control, which has since matured into a versatile framework for nonlinear and uncertain systems [17]. In IM drives, multiple studies report tangible benefits: lower steady-state error, improved transients, and reduced torque ripple. In particular, fuzzy controllers embedded within IFOC structures have routinely surpassed PI regulators at low speeds and under load perturbations [18,19,20,21]. Hybrid designs such as adaptive network-based fuzzy inference systems (ANFIS) further boost performance by marrying fuzzy inference with neural learning capabilities [22,23].

Optimization methods play an important role in tuning fuzzy gains and rule weights. Particle Swarm Optimization (PSO) and genetic algorithms (GAs) are frequently used to accelerate response, enhance power quality, and sharpen tracking accuracy [24,25,26,27]. Differential evolution (DE) offers another competitive option, combining fast convergence with stable adaptation [28,29].

Despite these gains, practical deployment often encounters a familiar bottleneck: rule-based growth. A typical 7 × 7 design demands 49 rules, inflating memory footprint and compute time in embedded, real-time settings [30,31]. To curb complexity, the literature proposes rule-reduction approaches, dominant-zone mapping, phase-plane simplification, and related heuristics [6,20]. Hardware-conscious implementations by Suetake et al. [32] and Talib et al. [20] demonstrate that compact fuzzy controllers can remain feasible at real-time rates when the rule base is pruned carefully.

Beyond rule count, researchers have explored richer inference engines. Type-2 and interval type-2 fuzzy systems better capture uncertainty but tend to be computationally heavy for fast drives [33,34]. Model-based strategies such as MPC and sliding-mode control (SMC) deliver high accuracy, yet they either demand substantial online optimization effort or suffer from chattering without careful smoothing [35,36]. Neural approaches (ANNs and ANFIS) remain attractive for their learning capability, though they require representative datasets and nontrivial training time [37]. More recently, fuzzy logic has been coupled with deep learning and reinforcement learning to enable real-time rule tuning, an approach gaining traction in renewables and MPPT contexts [38,39].

The scope of fuzzy control has also expanded to coordinated and multi-motor systems where synchronization and adaptive load sharing are essential [40,41,42]. Coordinated fuzzy schemes have improved torque balancing and transient efficiency in electric-vehicle drivetrains and conveyor networks. In parallel, reinforcement-learning-based fuzzy controllers show promise for online self-tuning of rules and membership functions by interacting with the plant and environment, a property well suited to evolving operating conditions [43,44]. For resource-limited hardware, ultra-compact realizations using lookup tables and other memory-efficient structures reduce latency and power draw without sacrificing control quality [45,46,47,48].

Recent developments in robust nonlinear control have extended conventional sliding-mode methods through adaptive higher-order and observer-assisted finite-time formulations. Barrier-function-based variable-gain super-twisting control can adjust the switching gain without prior knowledge of the disturbance derivative bound, thereby reducing gain overestimation while preserving finite-time convergence [49]. In parallel, finite-time nonlinear extended-state observers have been applied to permanent-magnet motor systems to estimate lumped disturbances and model uncertainties for robust feedback compensation [50]. Although these methods provide strong disturbance rejection and convergence properties, their nonlinear switching laws, observer dynamics, and associated gain-selection requirements may increase online implementation complexity compared with compact fuzzy-control structures.

Beyond electric drives, fuzzy control has also been applied to industrial plants and individual process subgroups in which accurate first-principles modeling is difficult and operating conditions vary substantially. A recent industrial example is the fuzzy-controlled feed-rate regulation strategy proposed for vibrating screens in mineral-processing plants, where feeder speeds are adjusted according to process-state information to mitigate overload conditions and improve operational stability [51]. This application illustrates the broader suitability of fuzzy inference for nonlinear industrial subsystems characterized by uncertain material flow, interacting operating constraints, and the need for interpretable real-time control actions.

Although reduced-rule and adaptive fuzzy controllers have both been reported, they have generally been developed as separate design directions. Reduced-rule approaches primarily seek to decrease inference and memory requirements through rule pruning, dominant-region selection, or simplified fuzzy mappings, but commonly retain fixed input–output scaling after deployment. Conversely, adaptive and optimization-assisted FLCs improve operating-point robustness by tuning gains, membership functions, or rule parameters, but may retain relatively dense rule bases or introduce additional online adaptation and optimization requirements. The specific contribution of this study is therefore not the isolated use of a nine-rule structure, PSO, or gain adaptation. Rather, it is their implementation-oriented co-design: a fixed 3 × 3 Mamdani rule base is combined with a bounded error-dependent output gain whose two governing parameters are optimized offline by PSO. Consequently, no population-based search, rule reconstruction, or membership-function update is required during real-time operation. The resulting controller is further formulated and analyzed in discrete time, with bounded-disturbance stability established using a Lyapunov framework and closed-loop behavior examined under modeled sampling delay, signal quantization, measurement noise, and fixed-step execution. This combination directly targets the performance–complexity gap between static reduced-rule FLCs and computationally heavier adaptive fuzzy schemes.

## 3. Methodology and System Design

This section elaborates on the comprehensive modeling framework, control design principles, simulation environment, and analytical foundations adopted to develop and validate the proposed minimal-rule adaptive fuzzy logic controller for high-performance induction motor (IM) drives.

### 3.1. Induction Motor Modeling

A three-phase squirrel-cage induction motor is modeled using dynamic equations in the d-q rotating reference frame (Park’s transformation). This modeling approach transforms the stator and rotor quantities into a synchronous reference frame to simplify time-varying parameters into steady-state components. The voltage equations for stator and rotor circuits are formulated, incorporating resistances, inductances, flux linkages, and electromagnetic interactions. These equations are implemented in MATLAB/Simulink R2024a, which provides a precise numerical platform for simulating motor dynamics under various control strategies. The model uses a 1.5 kW benchmark induction motor parameter set for comparative simulation. The parameters were not obtained through direct identification of a specific laboratory motor; therefore, the conclusions are limited to the investigated model. The specific electrical and mechanical parameters of the motor used in this study are listed in Table 1.

Note on the motor parameters: The inductance values in Table 1 were rechecked for numerical consistency. In the adopted model, *L*_s_ and *L*_r_ denote the total stator and rotor self-inductances, respectively, while *L*_m_ denotes the mutual inductance. The corresponding leakage inductances and magnetic coupling coefficient are given by

(1) $$L_{ls}=L_{s}-L_{m}=0.016\;H$$

(2) $$L_{lr}=L_{r}-L_{m}=0.016\;H$$

(3) $$k=L_{m}\;{\left(L_{s}\;L_{r}\right)}^{-1/2}=0.942$$

The parameter set is used as a benchmark simulation model and was not obtained from finite-element analysis or direct parameter identification of a specific physical motor.

### 3.2. Control System Architecture

The controller operates within an indirect field-oriented control (IFOC) framework. This strategy decouples torque and flux by aligning the rotor flux vector with the d-axis, enabling independent control of torque-producing (q-axis) and flux-producing (d-axis) currents. The control system includes an outer speed loop and an inner current loop, which are necessary to regulate rotor speed and torque dynamically. In this architecture, the speed reference is continuously compared with the actual rotor speed, and the resulting error signal is used as the primary input to the fuzzy logic controller. This layered control ensures enhanced tracking precision and better adaptability under variable load and speed conditions.

Under rotor-flux-oriented control, the electromagnetic torque is proportional to the torque-producing q-axis current and is expressed as

(4) $$T_{e}(k)=K_{t}(k)i_{q}(k)$$

where the instantaneous torque coefficient is

(5) $$K_{t}(k)=\frac{3}{2}p\frac{L_{m}}{L_{r}}\psi_{r}(k)$$

Here, p is the number of pole pairs, $L_{m}$ and $L_{r}$ are the mutual and rotor self-inductances, respectively, $\psi_{r}(k)$ is the rotor-flux magnitude, and $i_{q}(k)$ is the torque-producing stator-current component. This relation is used in the mechanically grounded stability analysis presented in Section 3.5.

### 3.3. Fuzzy Logic Controller Formulation

The fuzzy logic speed controller is designed in the discrete-time domain to align with the digital implementation requirements. It utilizes two input variables: the speed tracking error e(k) and its rate of change Δe(k).

The speed signals supplied to the outer-loop controller are expressed in revolutions per minute. Accordingly, the physical speed-tracking error is defined as

(6) $$e_{RPM}\left(k\right)=n_{ref}\left(k\right)-n_{r}\left(k\right)\;\;\left[RPM\right]$$

where $n_{ref}\left(k\right)$ and $n_{r}\left(k\right)$ denote the reference and measured rotor speeds in RPM, respectively. The corresponding error increment is

(7) $$\Delta e_{RPM}\left(k\right)=e_{RPM}\left(k\right)-e_{RPM}\left(k-1\right)\;\;\left[RPM/sample\right]$$

For use in the SI-unit mechanical model, the angular-speed error is obtained from

(8) $$e_{\omega }\left(k\right)=\omega_{ref}\left(k\right)-\omega_{r}\left(k\right)=\frac{2\pi }{60}e_{RPM}\left(k\right)\;\;\left[rad/s\right]$$

The controller inputs are formed from the RPM-domain quantities and mapped to the normalized interval [−1, 1] as

(9) $$e_{n\left(k\right)}=sat\left(K_{e}e_{RPM\left(k\right)},-1,1\right)$$

(10) $$\Delta e_{n\left(k\right)}=sat\left(K_{\Delta e}\Delta e_{RPM\left(k\right)},-1,1\right)$$

(11) $$K_{e}=\frac{1}{1440\;RPM},\;K_{\Delta e}=\frac{1}{1440\;RPM}\;\;\;\;\;\;$$

A minimal rule base consisting of nine fuzzy inference rules is employed by selecting three linguistic terms for each input variable. This reduced structure lowers the inference burden while retaining the directional resolution required for the normalized outer-loop speed dynamics considered in this study; the continuous membership-function overlap and adaptive output gain provide the intermediate control resolution that is not explicitly represented by additional linguistic sets.

The normalized variables defined above constitute the universes of discourse of the two fuzzy-controller inputs. The saturation operator is defined as

(12) $$sat\left(x,-1,1\right)=\min\left\{1,\max\left[-1,x\right]\right\}$$

Thus, a rated-speed error or an error increment equal in magnitude to the rated speed is mapped to the boundary of the normalized interval. The normalized fuzzy output is defined over the interval [−1, 1] and is subsequently scaled by the adaptive gain K(k) after defuzzification.

Three linguistic labels are assigned to each input and to the output: negative (N), zero (Z), and positive (P). Symmetric trapezoidal shoulder functions are used for N and P, while a triangular function is used for Z. For a generic normalized variable $x\in \left[-1,1\right]$, the membership functions are

(13) $$\mu_{N}\left(x\right)=trapmf\left(x;-1,-1,-0.5,0\right)$$

(14) $$\mu_{Z}\left(x\right)=trimf\left(x;-0.5,0,0.5\right)$$

(15) $$\mu_{P}\left(x\right)=trapmf\left(x;0,0.5,1,1\right)$$

The same breakpoints are used for $e_{n}$, $\Delta e_{n}$, and $u_{FLC}$. This symmetric parameterization provides full coverage of the normalized universe, 50% overlap between adjacent sets, and a monotonic control surface without discontinuities.

For reproducibility, Table 2 summarizes the physical ranges, normalized universes of discourse, and scaling factors used to map the speed error, error increment, and fuzzy-controller output into the normalized fuzzy domain.

All speed-domain performance values are reported in RPM, whereas angular speed in the mechanical model is expressed in rad/s. The conversion factor 2π/60 is applied whenever the controller-domain speed error is introduced into the SI-unit mechanical equations.

Based on the normalized variables defined in Table 2, Table 3 reports the complete membership-function specification, including the function type and numerical breakpoints assigned to the negative, zero, and positive linguistic sets. The same parameterization is applied to both normalized inputs and to the normalized fuzzy output.

The complete nine-rule inference matrix is presented in Table 4, where the rows represent the normalized speed-error sets, the columns represent the normalized error-increment sets, and each cell specifies the corresponding normalized fuzzy-output set. The rule base implements a monotonic PD-like control policy: when the error and its increment have the same sign, a strong corrective action is generated, whereas opposite signs weaken the output to limit overshoot as the speed trajectory approaches the reference.

Equivalently, the nine rules are as follows:

1. If $e_{n}$ is N and $\Delta e_{n}$ is N, then $u_{FLC}$ is N.

2. If $e_{n}$ is N and $\Delta e_{n}$ is Z, then $u_{FLC}$ is N.

3. If $e_{n}$ is N and $\Delta e_{n}$ is P, then $u_{FLC}$ is Z.

4. If $e_{n}$ is Z and $\Delta e_{n}$ is N, then $u_{FLC}$ is N.

5. If $e_{n}$ is Z and $\Delta e_{n}$ is Z, then $u_{FLC}$ is Z.

6. If $e_{n}$ is Z and $\Delta e_{n}$ is P, then $u_{FLC}$ is P.

7. If $e_{n}$ is P and $\Delta e_{n}$ is N, then $u_{FLC}$ is Z.

8. If $e_{n}$ is P and $\Delta e_{n}$ is Z, then $u_{FLC}$ is P.

9. If $e_{n}$ is P and $\Delta e_{n}$ is P, then $u_{FLC}$ is P.

The Mamdani inference mechanism uses the minimum operator for antecedent conjunction and implication, the maximum operator for rule aggregation, and centroid defuzzification. All rules have unit weight. The defuzzified normalized output is therefore

(16) $$u_{FLC}\left(k\right)=\frac{\int_{-1}^{1}z\;\mu_{agg}\left(z\right)\;dz}{\int_{-1}^{1}\mu_{agg}\left(z\right)\;dz}\;$$

and the signal applied to the torque-current reference is

(17) $$u\left(k\right)=K\left(k\right)u_{FLC}\left(k\right)$$

with $K\left(k\right)$ bounded as defined in Section 3.4.

The adequacy of the 3 × 3 rule structure is associated with the controlled operating envelope and the hierarchical IFOC architecture adopted in this study. The inner current loops regulate the flux- and torque-producing current components and compensate for the fast electrical dynamics; therefore, the outer fuzzy speed controller is not required to represent the complete nonlinear electromagnetic behavior of the induction motor. Its principal function is to determine the direction and relative intensity of the torque-current correction from the speed error and its temporal tendency. After normalization and saturation to the interval [−1, 1], each input is partitioned into three physically interpretable regions: negative, near-zero, and positive. Their Cartesian combination yields the nine operating situations required to distinguish acceleration, deceleration, convergence, divergence, and near-equilibrium behavior.

A larger number of linguistic partitions would increase local resolution but would also introduce additional rule transitions, memory requirements, and inference operations. In the proposed controller, the resolution normally supplied by intermediate fuzzy sets is partly recovered through the continuously varying membership grades and the bounded adaptive output gain. The membership-function overlap provides smooth interpolation between adjacent rule regions, whereas $K_{\left(k\right)}$ scales the control magnitude according to the instantaneous error without modifying the rule base. Thus, the nine rules determine the qualitative control direction, while the adaptive gain supplies the required quantitative variation over the considered speed and load range. The comparative results against the classical 49-rule FLC subsequently demonstrate that this compact partition preserves the required tracking and disturbance rejection performance under the operating scenarios defined in this study. Accordingly, the 3 × 3 structure should be interpreted as sufficient for the normalized operating envelope investigated herein, rather than as a universally optimal rule-base size for all induction motor ratings and operating regimes. The inference engine operates based on Mamdani’s method, and the center-of-gravity (COG) method is used for defuzzification to produce the normalized output $u_{FLC\left(k\right)}$.

### 3.4. Self-Tuning Adaptive Gain Mechanism

To improve transient performance without increasing the fuzzy rule-base size, the normalized fuzzy-controller output is scaled by a bounded adaptive gain. The gain law is formulated using the normalized speed error so that its numerical action is independent of whether the physical speed is expressed in RPM or rad/s:

(18) $$K\left(k\right)=K_{0}\left[1+\alpha \left|e_{n}\left(k\right)\right|\right]$$

where $K_{0}$ is the nominal output gain and α is a dimensionless sensitivity coefficient. Because the normalized error satisfies

(19) $$-1\leq e_{n}\left(k\right)\leq 1$$

it follows that

(20) $$0\leq \left|e_{n}\left(k\right)\right|\leq 1$$

and the adaptive gain is inherently bounded as

(21) $$K_{0}\leq K\left(k\right)\leq K_{0}\left(1+\alpha \right)$$

Accordingly,

(22) $$K_{min}=K_{0}$$

and

(23) $$K_{max}=K_{0}\left(1+\alpha \right)$$

Using the PSO-derived values $K_{0}=1.85$ and α=0.12, the gain bounds become

(24) $$K_{min}=1.85$$

and

(25) $$K_{max}=1.85\left(1+0.12\right)=2.072$$

Hence, throughout the normalized operating range,

(26) $$1.85\leq K\left(k\right)\leq 2.072$$

The normalized fuzzy input–output mapping is denoted by

(27) $$\Phi \left(e_{n}\left(k\right),\Delta e_{n}\left(k\right)\right)=u_{FLC}\left(k\right)$$

The adopted rule base and membership functions define a continuous and bounded nonlinear mapping over the normalized input domain. Let $\Omega_{n}$ denote the admissible portion of the normalized operating envelope, excluding the near-zero region, in which the fuzzy corrective action has the same sign as the normalized speed error. Within $\Omega_{n}$,

(28) $$e_{n}\left(k\right)\;\Phi \left(e_{n}\left(k\right),\Delta e_{n}\left(k\right)\right)\geq 0$$

For $e_{n}\left(k\right)\neq 0$, define the normalized local fuzzy slope as

(29) $$\rho_{n}\left(k\right)=\frac{\Phi \left(e_{n}\left(k\right),\Delta e_{n}\left(k\right)\right)}{e_{n}\left(k\right)}\;\;\;\;$$

Because the normalized fuzzy mapping is continuous and bounded over the compact admissible region, its local slope is assumed to satisfy

(30) $$0\leq \rho_{n}\left(k\right)\leq \rho_{n,max}$$

The physical controller output applied as the increment of the q-axis current reference is

(31) $$u\left(k\right)=K\left(k\right)u_{FLC}\left(k\right)=K\left(k\right)\Phi \left(e_{n}\left(k\right),\Delta e_{n}\left(k\right)\right)$$

To connect the dimensionless fuzzy mapping to the angular-speed error used in the induction motor mechanical model, the combined effects of RPM-domain normalization, RPM-to-rad/s conversion, the fuzzy mapping, and the adaptive gain are represented by an equivalent physical-domain sector slope $\kappa_{\omega }\left(k\right)$. The controller output is therefore written as

(32) $$u\left(k\right)=\kappa_{\omega }\left(k\right)e_{\omega }\left(k\right)+r_{0}\left(k\right)$$

where $r_{0}\left(k\right)$ is the bounded residual contribution associated with the near-zero fuzzy region. Outside that region, $r_{0}\left(k\right)=0$. The equivalent slope satisfies

(33) $$0\leq \kappa_{\omega }\left(k\right)\leq \kappa_{\omega ,max}$$

and the residual term satisfies

(34) $$\left|r_{0}\left(k\right)\right|\leq {\bar{r}}_{0}$$

The quantity $\kappa_{\omega ,max}$ is a bound on the complete controller slope seen by the mechanical speed-error dynamics. It incorporates the input normalization, the normalized fuzzy mapping, the bounded adaptive gain, and the conversion between the RPM-domain controller variables and the angular-speed error. This representation preserves the nonlinear two-input structure of the fuzzy controller while providing a dimensionally consistent bounded relation for the mechanical stability analysis.

### 3.5. Lyapunov Stability Analysis Based on the IM Mechanical Dynamics

The stability analysis is developed from the induction motor mechanical dynamics rather than from an isolated integrator approximation. In the synchronous d-q model, the rotor mechanical equation is

(35) $$J\frac{d\omega_{r}}{dt}=T_{e}-T_{L}-B\omega_{r}\;\;\;\;\;$$

where J is the total rotor-load inertia, B is the viscous-friction coefficient, $T_{L}$ is the load torque, and $T_{e}$ is the electromagnetic torque. Under rotor-flux-oriented control,

(36) $$T_{e}\left(k\right)=K_{t}\left(k\right)i_{q}\left(k\right)$$

with

(37) $$K_{t}\left(k\right)=\frac{3}{2}p\frac{L_{m}}{L_{r}}\psi_{r}\left(k\right)\;\;$$

where p is the number of pole pairs, $\psi_{r}\left(k\right)$ is the rotor-flux magnitude, and $i_{q}\left(k\right)$ is the torque-producing stator-current component. The inner flux and current loops are assumed to be stable and bandwidth-separated from the outer speed loop. Consequently, the torque coefficient is positive and bounded:

(38) $$0<K_{t,min}\leq K_{t}\left(k\right)\leq K_{t,max}\;\;$$

The analysis is performed in incremental variables around a constant-speed operating point. Define the angular-speed tracking error as

(39) $$e_{\omega }\left(k\right)=\omega_{ref}\left(k\right)-\omega_{r}\left(k\right)$$

where $e_{\omega }\left(k\right)$ is expressed in rad/s. Its relation to the RPM-domain controller error is

(40) $$e_{\omega }\left(k\right)=\frac{2\pi }{60}e_{RPM}\left(k\right)\;\;$$

Let $u\left(k\right)$ denote the controller-generated increment of the q-axis current reference. After subtracting the steady-state torque balance

(41) $$T_{e,0}=T_{L,0}+B\omega_{ref}\;\;\;$$

the incremental mechanical error dynamics can be written as

(42) $$J\frac{de_{\omega }}{dt}=-Be_{\omega }-K_{t}\left(t\right)u\left(t\right)+d_{m}\left(t\right)$$

where $d_{m}\left(t\right)$ collects the load-torque variation, q-axis current-tracking error, rotor-flux mismatch, and unmodeled mechanical effects. The disturbance is assumed to be bounded:

(43) $$\left|d_{m}\left(t\right)\right|\leq {\bar{d}}_{m}$$

Applying forward-Euler discretization with sampling period $T_{s}$ gives

(44) $$e_{\omega }\left(k+1\right)=\left(1-\frac{BT_{s}}{J}\right)e_{\omega }\left(k\right)-\frac{T_{s}}{J}K_{t}\left(k\right)u\left(k\right)+\frac{T_{s}}{J}d_{m}\left(k\right)\;\;\;\;\;\;\;\;\;\;\;\;$$

Using the equivalent-sector representation established in Section 3.4,

(45) $$u\left(k\right)=\kappa_{\omega }\left(k\right)e_{\omega }\left(k\right)+r_{0}\left(k\right)$$

with

(46) $$0\leq \kappa_{\omega }\left(k\right)\leq \kappa_{\omega ,max}$$

and

(47) $$\left|r_{0}\left(k\right)\right|\leq {\bar{r}}_{0}$$

Substitution yields

(48) $$e_{\omega }\left(k+1\right)=q\left(k\right)e_{\omega }\left(k\right)+w_{a}\left(k\right)$$

where

(49) $$q\left(k\right)=1-\frac{BT_{s}}{J}-\frac{T_{s}}{J}K_{t}\left(k\right)\kappa_{\omega }\left(k\right)\;\;$$

and

(50) $$w_{a}\left(k\right)=\frac{T_{s}}{J}\left[d_{m}\left(k\right)-K_{t}\left(k\right)r_{0}\left(k\right)\right]\;\;\;\;$$

Because $d_{m}\left(k\right)$, $K_{t}\left(k\right)$, and $r_{0}\left(k\right)$ are bounded, the augmented disturbance is also bounded:

(51) $$\left|w_{a}\left(k\right)\right|\leq {\bar{w}}_{a}$$

where

(52) $${\bar{w}}_{a}=\frac{T_{s}}{J}\left({\bar{d}}_{m}+K_{t,max}{\bar{r}}_{0}\right)\;\;\;$$

Define the combined feedback coefficient

(53) $$\mu_{\omega }\left(k\right)=K_{t}\left(k\right)\kappa_{\omega }\left(k\right)$$

It satisfies

(54) $$0\leq \mu_{\omega }\left(k\right)\leq \mu_{\omega ,max}$$

with

(55) $$\mu_{\omega ,max}=K_{t,max}\kappa_{\omega ,max}$$

The sampled closed-loop coefficient can therefore be written as

(56) $$q\left(k\right)=1-\frac{BT_{s}}{J}-\frac{T_{s}}{J}\mu_{\omega }\left(k\right)\;$$

Because $q\left(k\right)$ is affine in $\mu_{\omega }\left(k\right)$, its maximum absolute value over the admissible interval occurs at an endpoint. A sufficient worst-case contraction bound is therefore

(57) $$\bar{q}=max\left\{\left|1-\frac{BT_{s}}{J}\right|,\left|1-\frac{BT_{s}}{J}-\frac{T_{s}}{J}K_{t,max}\kappa_{\omega ,max}\right|\right\}<1\;\;\;\;\;\;\;\;\;\;\;\;$$

Consider the mechanical-energy-scaled Lyapunov candidate

(58) $$V\left(k\right)=\frac{J}{2}e_{\omega }^{2}\left(k\right)\;\;\;\;\;\;$$

Its forward difference is

(59) $$\Delta V\left(k\right)=\frac{J}{2}\left\{{\left[q\left(k\right)e_{\omega }\left(k\right)+w_{a}\left(k\right)\right]}^{2}-e_{\omega }^{2}\left(k\right)\right\}$$

Expanding and using $\left|q\left(k\right)\right|\leq \bar{q}$ and $\left|w_{a}\left(k\right)\right|\leq {\bar{w}}_{a}$ gives

(60) $$\Delta V\left(k\right)\leq -\frac{J}{2}\left(1-{\bar{q}}^{2}\right)e_{\omega }^{2}\left(k\right)+J\bar{q}{\bar{w}}_{a}\left|e_{\omega }\left(k\right)\right|+\frac{J}{2}{\bar{w}}_{a}^{2}\;$$

For any η>0, Young’s inequality gives

(61) $$J\bar{q}{\bar{w}}_{a}\left|e_{\omega }\left(k\right)\right|\leq \frac{J\eta }{2}e_{\omega }^{2}\left(k\right)+\frac{J{\bar{q}}^{2}}{2\eta }{\bar{w}}_{a}^{2}$$

Therefore,

(62) $$\Delta V\left(k\right)\leq -\frac{J}{2}\left(1-{\bar{q}}^{2}-\eta \right)e_{\omega }^{2}\left(k\right)+\frac{J}{2}\left(1+\frac{{\bar{q}}^{2}}{\eta }\right){\bar{w}}_{a}^{2}\;\;$$

Selecting

(63) $$0<\eta <1-{\bar{q}}^{2}$$

and defining

(64) $$\lambda =\frac{J}{2}\left(1-{\bar{q}}^{2}-\eta \right)>0$$

and

(65) $$\gamma_{a}=\frac{J}{2}\left(1+\frac{{\bar{q}}^{2}}{\eta }\right){\bar{w}}_{a}^{2}\;$$

yields

(66) $$\Delta V\left(k\right)\leq -\lambda e_{\omega }^{2}\left(k\right)+\gamma_{a}$$

Thus, within the stated admissible sector, the derived inequality establishes uniform ultimate boundedness under bounded disturbances whenever the sufficient contraction condition $\bar{q}<1$ is satisfied. In the disturbance-free case, for which ${\bar{w}}_{a}=0$, the same condition yields asymptotic convergence of the angular-speed tracking error to zero.

The result is a sufficient conditional stability criterion. Because $\kappa_{\omega ,max}$ was not numerically identified from the implemented fuzzy surface, the analysis should be interpreted as a conditional analytical guarantee rather than a numerical verification of the contraction margin.

### 3.6. Parameter Optimization Using Particle Swarm Optimization (PSO)

To eliminate the subjectivity of trial-and-error tuning and to fully exploit the potential of the proposed controller, the Particle Swarm Optimization (PSO) algorithm was employed to determine the best-performing values identified within the adopted PSO configuration of the nominal gain $k_{0}$ and the dimensionless normalized-error sensitivity coefficient α.

PSO is a population-based stochastic optimization technique inspired by the social behavior of bird flocking. In this study, the optimization objective is to minimize the Integral of Time-weighted Absolute Error (ITAE), which balances the trade-off between rise time and overshoot. The objective function J is defined as

(67) $$J=\int_{0}^{T_{sim}}t\;\times \left|e\left(t\right)\right|dt\;\;\;$$

The optimization problem is formulated as follows: Minimize $J\left(k_{0},\alpha \right).$ Subject to $0.1\leq k_{0}\leq 5.0$ and 0.01≤α≤1.0.

**Figure 1:**
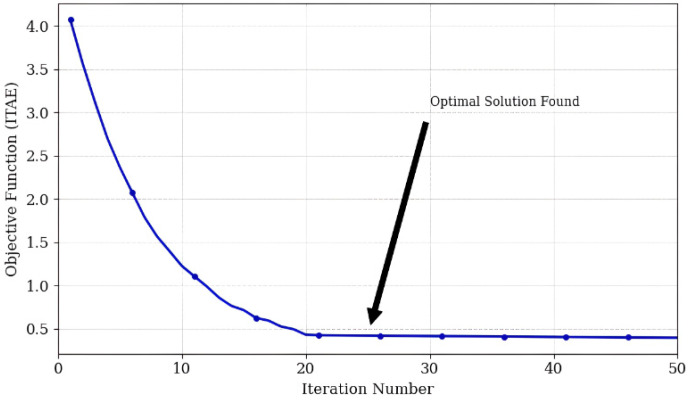
Convergence history of the best ITAE value obtained during the PSO search.

The PSO algorithm was configured with a swarm size of 20 particles and run for 50 iterations. Figure 1 illustrates the convergence of the best objective-function value obtained during the PSO run. The cost decreases rapidly during the early iterations and reaches a stable best-found solution after approximately 25 iterations. Because PSO is a stochastic metaheuristic, this convergence behavior indicates numerical stabilization of the best solution identified within the prescribed search bounds and computational budget, rather than a formal proof of global optimality. The best-performing parameter set obtained within the adopted PSO configuration and subsequently used in this study is listed in Table 5.

**Figure 2:**
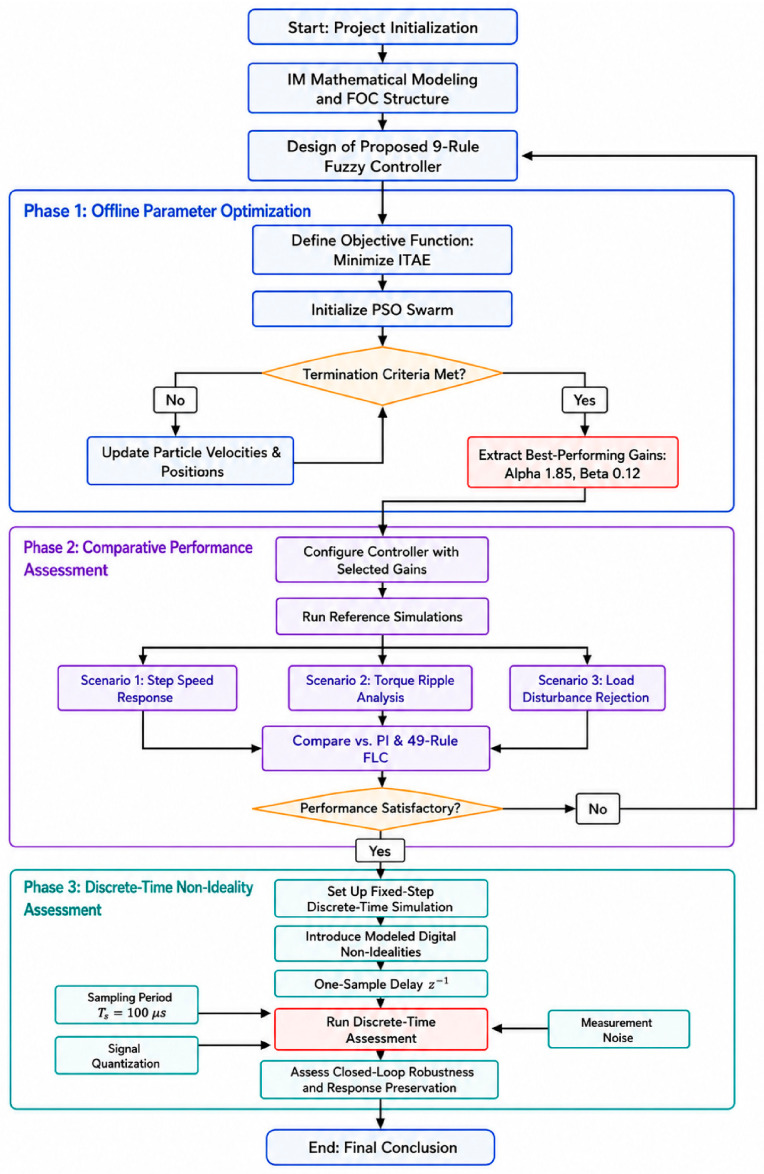
Comprehensive flowchart of the proposed research methodology, detailing the offline PSO parameter optimization, comparative performance analysis, and the discrete-time assessment under modeled digital non-idealities.

To provide a holistic view of the proposed control strategy, the systematic design procedure is illustrated in Figure 2. The flowchart summarizes the three-phase methodology adopted in this study: offline PSO-based parameter tuning of the proposed controller within the prescribed search bounds and computational budget (Phase 1), comparative performance assessment against the baseline controllers (Phase 2), and an additional fixed-step discrete-time simulation under modeled measurement noise, signal quantization, and a one-sample delay (Phase 3).

## 4. Simulation Setup and Reproducibility

The proposed control scheme is evaluated through comprehensive simulation studies conducted in the MATLAB/Simulink environment. To ensure consistent numerical evaluation of digital control effects, all control loops are implemented in discrete time.

The simulations were performed using a fixed-step discrete solver with a sampling period of Ts =100 μs. This sampling period was adopted as a fixed numerical control interval and was applied identically to all controllers.

Zero-mean Gaussian white noise with a standard deviation of 1.5 RPM and a variance of 2.25 RPM^2^ was added to the rotor speed feedback. The discrete-time disturbance test applied a load-torque step from 0.5 to 1.2 Nm at t = 0.6 s. A uniform 12-bit quantizer was used over the speed range [−1440, 1440] RPM, corresponding to a resolution of approximately 0.703 RPM/LSB. These non-idealities were applied identically to all controllers.

For the additional discrete-time assessment, the control algorithms are executed within a fixed-step simulation environment while accounting for the following modeled digital effects:

- One-sample computational delay ($z^{-1}$);
- Signal quantization effects;
- Fixed execution order of control tasks.

These modeled effects do not constitute execution of generated C/C++ code in an SIL or target-processor environment. No predictive, phase-lead, or explicit delay-compensation mechanism was applied. The one-sample delay was introduced directly into the control loop as an uncompensated modeled latency to evaluate the sensitivity of the closed-loop response to this non-ideality.

To ensure a fair and consistent comparison, all controllers (PI, classical 49-rule FLC, and the proposed 9-rule self-tuning FLC) are evaluated under identical simulation conditions, including the following:

- Identical sampling time;
- Identical disturbance and noise profiles;
- Identical reference trajectories and load conditions.

The baseline controllers were tuned independently before the final comparative tests. The PI controller was adjusted iteratively to obtain a stable and well-damped speed response with negligible overshoot, reduced settling time, and acceptable steady-state accuracy. The classical 49-rule FLC was implemented using a conventional two-input Mamdani structure with seven linguistic terms for both the speed error and the change in speed error. Its input and output scaling factors were adjusted offline through iterative response-based tuning over the investigated operating range, while the membership-function shapes and the complete 49-rule inference matrix remained fixed. The classical FLC was not optimized using the PSO–ITAE procedure adopted for the proposed controller and is therefore considered a conventionally tuned full-rule baseline.

The conventional PI controller and the classical 49-rule FLC were tuned independently before the final comparative tests, after which their parameters were kept fixed. The PI controller was adjusted iteratively to obtain a stable and well-damped response under the prescribed torque-current-reference constraint. The 49-rule FLC was implemented as a conventional two-input Mamdani controller with seven linguistic sets per input and a fixed 49-rule matrix. Its input and output scaling factors were adjusted offline through iterative response-based tuning, whereas its membership functions and rule base remained fixed. The proposed nine-rule FLC, by contrast, used PSO-based offline tuning of the nominal gain and normalized-error sensitivity coefficient, followed by bounded online scalar-gain adaptation. Consequently, the comparison was performed under identical simulation conditions but not under identical offline optimization procedures.

After the offline tuning stage, the PI and classical 49-rule FLC parameters were fixed and retained unchanged throughout all comparative simulations. No online optimization, rule modification, or membership-function retuning was applied to either baseline controller. All controllers were evaluated using the same induction motor model, sampling period, solver configuration, initial conditions, reference trajectories, load disturbances, actuator limits, measurement-noise profile, signal quantization, and modeled delay. The reported comparison therefore represents performance under identical test conditions, while the difference in the offline tuning procedures is explicitly acknowledged.

All simulation parameters, controller settings, and performance metrics were kept consistent throughout the study to support reproducibility of the reported results. The adopted fixed-step simulation framework was used to examine the controller responses under the specified sampling, noise, quantization, and delay conditions. This assessment does not establish processor-level execution feasibility or embedded readiness. The principal simulation parameters are summarized in Table 6.

### Performance Metrics and Evaluation Criteria

To ensure an objective and quantitative performance comparison among the evaluated controllers, a set of standard time-domain and energy-based performance metrics is employed. All metrics are computed under identical operating conditions and over predefined evaluation intervals.

Settling Time: The settling time is defined as the time required for the rotor speed response to remain within ±2% of its steady-state value following a reference change or an external disturbance.

Post-Transient Mean Absolute Tracking Error: The residual tracking performance is quantified using the mean absolute speed error over a finite evaluation window selected after the principal transient response. It is defined as

(68) $$e_{MAE,posts}=\left(\frac{1}{N}\right)\sum_{k=k_{0}}^{k_{0}+N}\left|e\left(k\right)\right|\;\;\;\;\;\;\;\;\;\;\;$$

where $k_{0}$ denotes the beginning of the post-transient evaluation window and N is the number of samples included in the calculation. This finite-window metric represents the average residual tracking deviation and should not be interpreted as the exact asymptotic steady-state offset of the controller.

Torque Ripple: Torque ripple is quantified using the peak-to-peak variation of the electromagnetic torque during steady-state operation. It is defined as

(69) $$Torque\;Ripple=T_{max}-T_{min}$$

This metric reflects the oscillatory behavior of the electromagnetic torque and its impact on mechanical stress and acoustic noise.

Control Effort: The control effort is evaluated using an energy-based criterion computed as the discrete-time sum of the squared control signal:

(70) $$J_{u}=\sum_{k=0}^{N}u^{2}(k)\;$$

This metric provides a quantitative measure of the actuation energy required by each controller and enables a fair comparison of control efficiency.

Algorithmic Complexity: The controller complexity is compared using the rule-base size and the number of fuzzy inference operations required per control update. Host-computer execution times are not used as evidence of embedded feasibility because they depend on the MATLAB execution environment and do not represent target-processor timing, interrupt latency, memory access, or peripheral overhead. Processor-specific execution feasibility therefore remains outside the validation scope of the present study.

## 5. Results and Discussion

This section presents and discusses the simulation results obtained under the reference and modeled digitally non-ideal test conditions. The performance is evaluated comparatively against a conventional PI controller and a classical 49-rule fuzzy logic controller under identical operating conditions.

### 5.1. Speed Response Analysis

To evaluate the dynamic speed tracking performance of the proposed minimal-rule fuzzy logic controller, a step change in the reference speed was applied, and the responses of three control strategies were comparatively analyzed: a conventional PI controller, a classical 49-rule fuzzy logic controller, and the proposed 9-rule self-tuning FLC.

**Figure 3:**
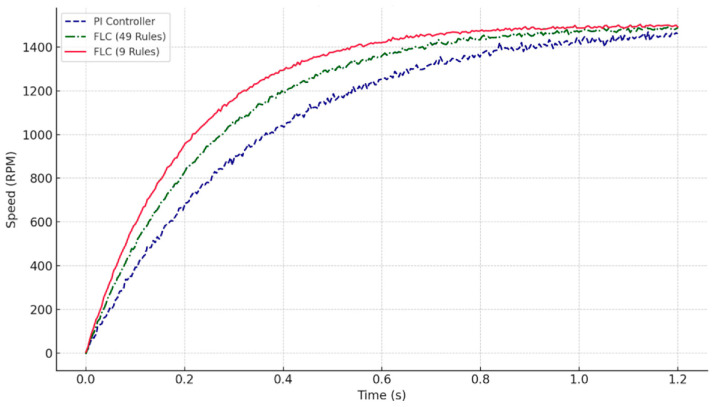
Speed response comparison under step reference input (PI, 49-rule FLC, and 9-rule FLC).

Figure 3 compares the rotor speed responses following a reference step from 0 to 1440 RPM. The conventional PI controller exhibits a relatively slow transient response, characterized by a rise time of about 0.21 s and a settling time close to 0.35 s. Over the selected post-transient evaluation window, the PI controller exhibits a mean absolute tracking error of approximately 18 RPM. This value represents the finite-window residual tracking deviation used for comparative evaluation and should not be interpreted as the asymptotic steady-state offset of the PI controller. The comparatively larger value is associated with the slower convergence observed within the adopted simulation interval.

The classical 49-rule fuzzy logic controller shows a clear improvement in transient performance. The rise time is reduced to around 0.18 s, while the settling time decreases to approximately 0.28 s. Moreover, the post-transient mean absolute tracking error decreases to 8 RPM. This improvement can be attributed to the richer inference capability provided by the larger rule base, which enables smoother control action and better handling of nonlinearities.

The proposed nine-rule self-tuning fuzzy logic controller achieves the fastest and most accurate response among the evaluated strategies. The rise time is reduced to 0.15 s, and the settling time reaches approximately 0.26 s, while the post-transient mean absolute tracking error is limited to 4 RPM. Despite the significantly reduced number of fuzzy rules, the adaptive gain mechanism allows the controller to respond aggressively during large speed deviations and gradually reduce the control gain as the error diminishes. This behavior results in rapid convergence without introducing overshoot or oscillatory effects.

From a quantitative perspective, the proposed controller reduces the settling time by approximately 25.7% compared to the PI controller and by 7.1% relative to the classical 49-rule fuzzy controller. These results demonstrate that a carefully designed reduction in fuzzy rule complexity, when combined with an effective self-tuning gain strategy, can preserve and even enhance dynamic performance. Such characteristics provide a favorable basis for future hardware-oriented evaluation, although embedded feasibility is not established by the present simulation results.

### 5.2. Torque Ripple Evaluation

Electromagnetic torque ripple is a key indicator of control quality in motor drive systems, as pronounced torque oscillations are directly associated with mechanical vibration, acoustic noise, and increased stress on drivetrain components. To assess the torque smoothness achieved by each control strategy, the electromagnetic torque response is analyzed under nominal speed and load conditions.

**Figure 4:**
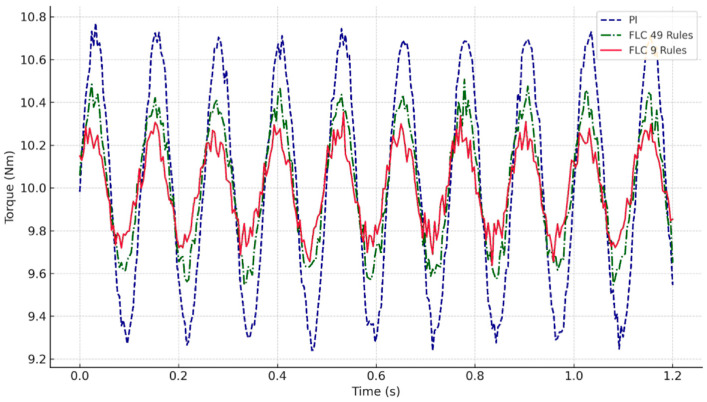
Electromagnetic torque ripple comparison for different controllers.

Figure 4 illustrates the instantaneous electromagnetic torque waveforms obtained using the conventional PI controller, the classical 49-rule fuzzy logic controller, and the proposed 9-rule self-tuning fuzzy controller. Clear differences in torque fluctuation patterns can be observed among the three control approaches.

The PI-controlled drive exhibits pronounced high-frequency torque oscillations, resulting in a peak-to-peak ripple of approximately 0.85 Nm. This behavior reflects the inherent limitations of fixed-parameter PI control, where abrupt variations in the control signal can occur during dynamic regulation, leading to overshoot and oscillatory torque behavior.

The classical 49-rule fuzzy logic controller significantly improves torque smoothness, reducing the peak-to-peak ripple to about 0.38 Nm. This improvement can be attributed to the richer inference capability provided by the larger rule base, which yields a smoother control surface and mitigates abrupt changes in the torque-producing current.

Despite employing a substantially reduced rule base, the proposed nine-rule adaptive fuzzy logic controller achieves a torque ripple amplitude of approximately 0.44 Nm. Although this value is marginally higher than that of the full-rule fuzzy controller (by about 15.7%), it remains notably lower than the ripple observed with PI control. The adaptive gain mechanism plays a crucial role in this performance by moderating sudden control actions during transient intervals while preserving adequate responsiveness.

These results indicate that a carefully designed minimal-rule fuzzy controller, when complemented by an adaptive gain strategy, can deliver torque smoothness comparable to that of a significantly more complex fuzzy controller. This balance between torque quality and reduced rule-base complexity highlights the controller’s favorable simulation-level performance–complexity trade-off.

### 5.3. Speed Error Dynamics

The evolution of the speed error over time provides a clear indication of a controller’s capability to accurately track the reference speed during both transient and steady-state operation. In applications where precise speed regulation is required, rapid error attenuation and minimal steady-state deviation are essential performance characteristics.

**Figure 5:**
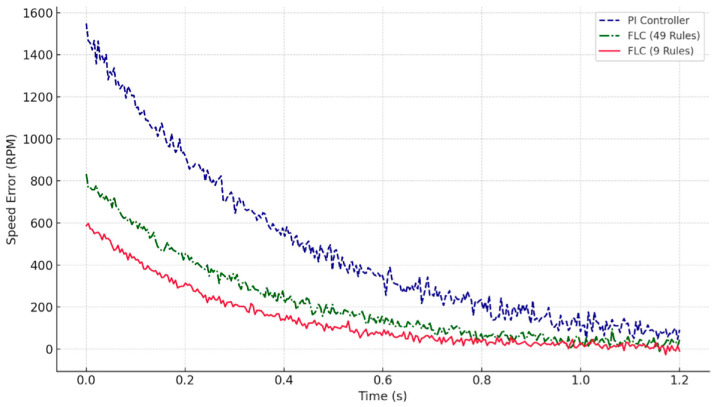
Instantaneous speed error dynamics comparison.

Figure 5 illustrates the instantaneous speed error profiles corresponding to the PI controller, the classical 49-rule fuzzy logic controller, and the proposed 9-rule adaptive fuzzy controller following a reference speed step. Distinct differences in error magnitude and convergence behavior can be observed among the three control strategies.

The PI-controlled system exhibits the largest speed error throughout the response interval. Immediately after the reference change, the error exceeds 25 RPM and decays slowly, requiring approximately 0.35 s to settle within a narrow tolerance band. This behavior reflects the inherent limitations of fixed-gain PI control, which lacks the ability to adjust its corrective action in response to rapidly changing error dynamics.

The classical 49-rule fuzzy logic controller provides a substantial improvement in error regulation. The peak error is reduced to around 6.3 RPM, and the convergence time decreases to approximately 0.29 s. This enhanced performance is associated with the rule-based inference mechanism of the fuzzy controller, which allows more context-aware correction compared to linear control.

The proposed nine-rule adaptive fuzzy logic controller achieves the most favorable error dynamics among all evaluated approaches. The peak speed error is limited to only 2.4 RPM, and the fastest convergence is obtained within approximately 0.23 s. Despite the significantly reduced rule base, the adaptive gain scaling mechanism enables the controller to increase the control effort during large deviations and gradually attenuate it as the error diminishes, ensuring rapid convergence without compromising steady-state stability.

These observations indicate that error convergence in the proposed controller is governed primarily by adaptive modulation of the control gain rather than by rule density alone. The self-tuning mechanism effectively compensates for the reduced inference resolution, resulting in enhanced tracking accuracy and smoother steady-state behavior.

Moreover, the reduced post-transient mean absolute tracking error indicates improved tracking accuracy under the investigated simulation conditions. This result should not be interpreted as evidence of robustness to untested motor-parameter variations, which requires dedicated rotor-resistance and inertia sensitivity studies. Overall, the results demonstrate that low-complexity fuzzy controllers, when augmented with adaptive features, can deliver high-precision speed regulation while exhibiting a structurally compact control architecture that may be considered for future evaluation in resource-constrained motor-drive platforms.

### 5.4. Control Signal Behavior

The behavior of the control signal provides direct insight into the quality and practicality of the control strategy, particularly in electric drive systems where excessive control activity may lead to increased switching losses, thermal stress, and accelerated wear of both mechanical and power electronic components. Consequently, a smooth and well-regulated control effort is a key indicator of a robust and implementation-friendly controller.

**Figure 6:**
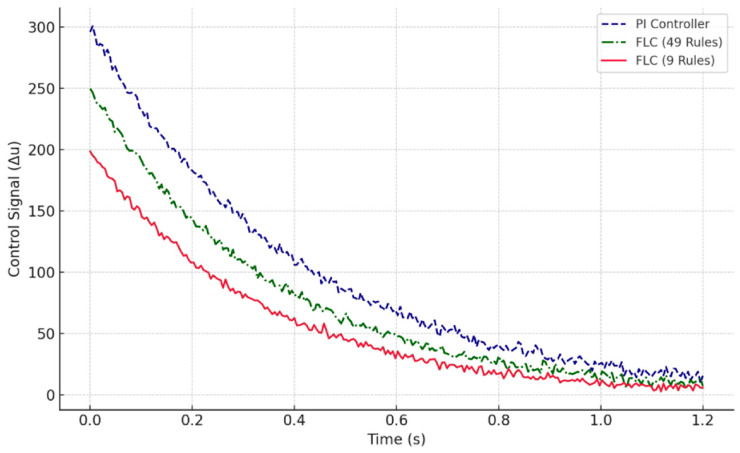
Control signal (Δu) output comparison for PI, 49-rule FLC, and 9-rule FLC.

Figure 6 illustrates the control signal responses generated by the PI controller, the classical 49-rule fuzzy logic controller, and the proposed 9-rule adaptive fuzzy controller following a step change in the reference speed. Clear differences in the magnitude and smoothness of the control actions can be observed among the three strategies.

The conventional PI controller produces sharp and high-amplitude control variations, especially during the initial transient interval. These abrupt changes indicate an overreactive control behavior with limited inherent damping, which may translate into torque pulsations and increased mechanical and electrical stress in practical drive systems.

The classical 49-rule fuzzy logic controller exhibits smoother control transitions compared to the PI controller, reflecting the benefits of rule-based nonlinear inference. However, despite its larger rule base, noticeable micro-oscillations persist in the control signal. This behavior suggests a degree of redundancy within the rule set, where frequent switching between neighboring inference rules can introduce unnecessary control fluctuations.

In contrast, the proposed nine-rule adaptive fuzzy logic controller generates the most stable and continuous control signal throughout both transient and steady-state operation. The control effort remains relatively low in amplitude and evolves smoothly over time, indicating improved regulation of the actuation process. This behavior is primarily attributed to two complementary design features. First, the simplified 3 × 3 rule structure reduces excessive rule switching, resulting in a more predictable and gradual control response. Second, the adaptive gain tuning mechanism dynamically adjusts the control increment according to the magnitude of the speed error, enabling stronger corrective action during transients while suppressing unnecessary control activity as steady-state conditions are approached.

As a result, the proposed controller achieves a well-balanced control signal that enhances energy efficiency and reduces switching stress and electromagnetic interference in inverter-based drive systems. These characteristics further support further hardware-oriented evaluation of the proposed approach for industrial motor-drive applications, where reliability and long-term operational efficiency are critical.

### 5.5. Robustness to Load Variation

Robustness against load disturbances is a key requirement for motor drive controllers intended for real-world industrial operation, where mechanical loads may vary abruptly due to changes in operating conditions, coupling dynamics, or external forces. An effective controller must, therefore, be capable of quickly compensating for such disturbances while maintaining stable speed regulation.

**Figure 7:**
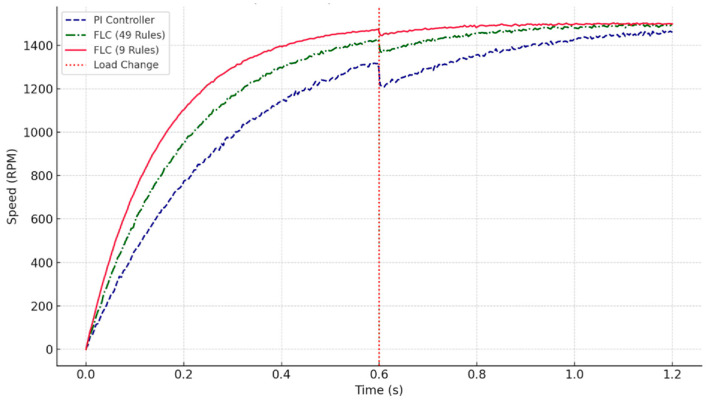
Motor speed response under sudden load variation (robustness evaluation).

Figure 7 illustrates the rotor speed responses of the induction motor when a step increase in load torque is applied, rising from 0.5 Nm to 1.2 Nm at t=0.6 s. This disturbance introduces a sudden torque demand that momentarily reduces the rotor speed and challenges the controller’s ability to restore the reference value.

The conventional PI controller exhibits the weakest disturbance rejection performance. Following the load increase, the rotor speed drops by nearly 60 RPM, and the recovery process is relatively slow, with the system requiring approximately 0.61 s to reestablish steady operation. This behavior reflects the limited adaptability of fixed-gain PI control, which cannot dynamically adjust its response to abrupt changes in load conditions.

The classical 49-rule fuzzy logic controller shows a noticeable improvement in robustness. The speed deviation is reduced to around 35 RPM, and the recovery time shortens to approximately 0.38 s. The larger rule base enables the controller to interpret a wider range of error patterns, resulting in more effective corrective action during the disturbance interval.

The proposed nine-rule adaptive fuzzy logic controller demonstrates the highest level of robustness among the evaluated strategies. The speed drop is limited to only 22 RPM, and the system recovers rapidly within about 0.30 s. This enhanced performance arises from the combined effect of adaptive gain scaling and streamlined rule inference. When a large error is detected following the load change, the adaptive gain temporarily increases the control effort, accelerating recovery. At the same time, the reduced overlap between fuzzy rules promotes focused and decisive control actions, avoiding unnecessary oscillations.

Importantly, this superior disturbance rejection is achieved with a substantially lower computational burden compared to the classical fuzzy controller. These results identify the proposed controller as a structurally compact candidate for future embedded evaluation. Processor-level suitability cannot be established without target-specific execution-time, memory-footprint, HIL, and experimental measurements.

### 5.6. Quantitative Comparison and Computational Analysis

A comprehensive assessment of motor drive controllers requires not only qualitative inspection of time-domain responses, but also a quantitative comparison across multiple performance indicators and computational resource requirements. In this section, the results obtained in the previous analyses are consolidated and evaluated using numerical metrics and visual benchmarking tools in order to highlight both control effectiveness and implementation efficiency.

**Figure 8:**
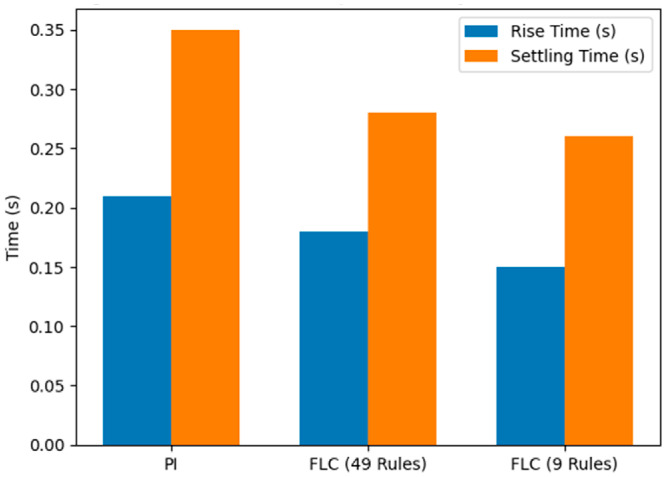
Bar chart comparing rise time and settling time for different controllers.

**Figure 9:**
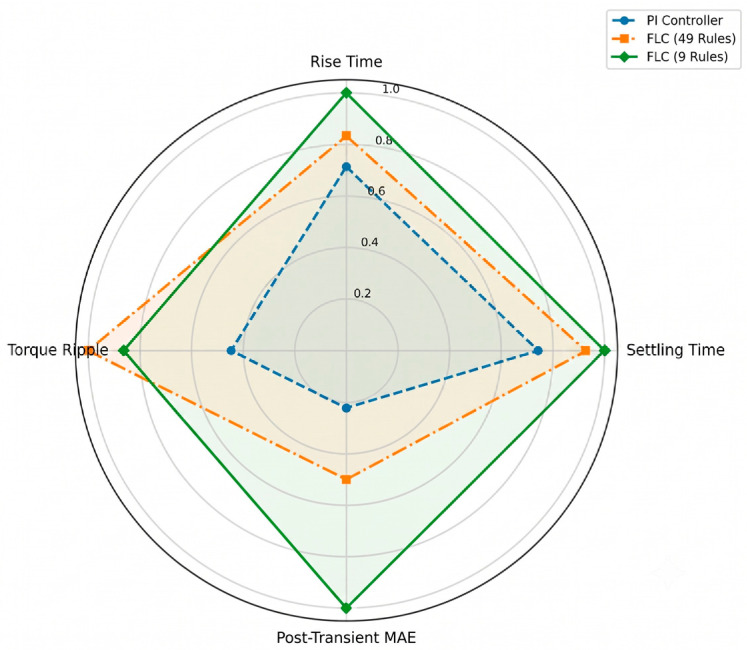
Normalized radar-chart comparison based on simulation-derived control-performance metrics reported in Table 7 and the torque-ripple results of Section 5.2. Higher normalized scores indicate better performance; each lower-is-better metric was normalized as the ratio of the best observed value to the corresponding controller value.

Figure 8 and Figure 9 provide a compact visual summary of the comparative performance of the three controllers. Figure 8 presents a bar-chart comparison of the principal time-domain metrics, including rise time and settling time, under identical step-reference conditions. Figure 9 extends the comparison through a normalized radar chart based only on simulation-derived control-performance indicators, including rise time, settling time, post-transient mean absolute tracking error, and torque-ripple magnitude. Processor utilization, execution time, and memory usage are not included because these quantities were not measured on a target embedded platform.

The quantitative results confirm that the conventional PI controller delivers the weakest overall performance. Because the baseline-controller parameters were fixed before the final evaluation and identical operating, disturbance, and numerical conditions were imposed on all controllers, the following comparison reflects differences in closed-loop behavior rather than post hoc retuning for individual test cases. It exhibits the longest rise time of 0.21 s and the largest post-transient mean absolute tracking error, measured as 18 RPM over the selected evaluation window. While the PI controller benefits from low computational requirements, its response is characterized by a sluggish convergence to the reference speed, making it less suitable for applications involving rapid operating point changes.

The classical 49-rule fuzzy logic controller achieves a clear improvement in dynamic precision. The rise time is reduced to 0.18 s, and the post-transient mean absolute tracking error decreases to 8 RPM. Its 49-rule inference structure, however, requires up to 49 fuzzy-rule evaluations per control update, representing the highest structural fuzzy-inference complexity among the evaluated controllers.

The proposed nine-rule self-tuning fuzzy logic controller demonstrates the most balanced performance among the evaluated strategies. It achieves the shortest rise time of 0.15 s and the smallest post-transient mean absolute tracking error of 4 RPM, while retaining a smooth response with negligible overshoot. Structurally, the controller requires up to nine fuzzy-rule evaluations per control update and one bounded scalar-gain update. Relative to the classical 49-rule FLC, this corresponds to an 81.6% reduction in the maximum number of fuzzy-rule evaluations per update. This reduction indicates lower structural algorithmic complexity but does not establish processor-level execution time, memory usage, or embedded implementation feasibility.

Figure 9 summarizes the comparative simulation-level performance using normalized control metrics only. The radar-chart axes represent rise time, settling time, post-transient mean absolute tracking error, and torque-ripple magnitude. The proposed controller provides the most balanced control-performance profile among the evaluated strategies. The chart does not include CPU utilization, execution time, or memory consumption because no target-processor measurements were performed.

The numerical performance metrics summarized in Table 7 reinforce these observations. Across all evaluated indicators, the proposed controller consistently outperforms the PI controller and closely approximates or exceeds the performance of the classical fuzzy controller with significantly fewer rules. This comparison also provides an empirical sufficiency check for the selected 3 × 3 partition over the investigated operating envelope. Increasing the rule base from 9 to 49 produces only limited improvement in torque-ripple attenuation, whereas the proposed controller achieves shorter rise and settling times, a lower post-transient mean absolute tracking error, and substantially fewer maximum fuzzy-rule evaluations per control update. The results therefore indicate that additional linguistic partitions are not required to represent the dominant outer-loop speed-error dynamics considered in the present tests. Table 7 summarizes the time-domain performance metrics, whereas Table 8 compares the structural algorithmic characteristics of the evaluated controllers. MATLAB Profiler timings are omitted because host-computer measurements cannot be interpreted as execution times on a microcontroller or DSP. Because no target DSP or microcontroller implementation was performed, hardware-dependent quantities such as worst-case execution time, interrupt latency, memory usage, and processor utilization remain to be verified experimentally.

The PI controller does not require fuzzy-rule evaluation and therefore has the lowest structural complexity. The classical 49-rule FLC requires up to 49 rule evaluations per control update, whereas the proposed controller requires up to nine rule evaluations and one scalar-gain update. Relative to the classical FLC, the proposed structure reduces the maximum number of fuzzy-rule evaluations by approximately 81.6%. These quantities describe structural algorithmic complexity only. Actual execution time, worst-case latency, memory footprint, and processor utilization must be established through implementation on a specified DSP or microcontroller.

Overall, the results identify the proposed controller as a structurally compact candidate for future embedded evaluation. The present simulation study does not establish target-processor execution time, worst-case latency, memory footprint, processor utilization, or practical deployment capability. These aspects require generated-code SIL, Hardware-in-the-Loop testing, and implementation on a specified embedded platform.

The structural algorithmic characteristics of the three controllers are summarized in Table 8.

Table 8 shows that the proposed controller requires up to 9 fuzzy-rule evaluations per update, compared with up to 49 for the classical FLC. The additional online operation is limited to a scalar gain update. These quantities indicate reduced algorithmic complexity; however, actual execution time, memory footprint, and processor utilization must be measured on the selected hardware platform.

### 5.7. Performance Under Time-Varying Random Load Torque

To further examine the robustness of the proposed minimal-rule adaptive fuzzy logic controller, an additional test scenario involving time-varying and stochastic load torque disturbances was conducted. This case represents practical operating conditions encountered in industrial motor drive systems, where load torque may fluctuate unpredictably due to process variations, mechanical irregularities, or environmental influences.

In this test, a bounded pseudo-random torque disturbance was applied to the motor shaft. The disturbance signal was generated by filtering a white-noise sequence through a low-pass filter with a cutoff frequency of 5 Hz, resulting in a smooth yet rapidly varying torque profile with an amplitude limited to ± 0.7 Nm. This configuration ensures that the disturbance remains physically realistic while introducing continuous and non-deterministic load variations. All simulations were carried out under identical conditions for the conventional PI controller, the classical 49-rule fuzzy logic controller, and the proposed 9-rule adaptive fuzzy logic controller.

**Figure 10:**
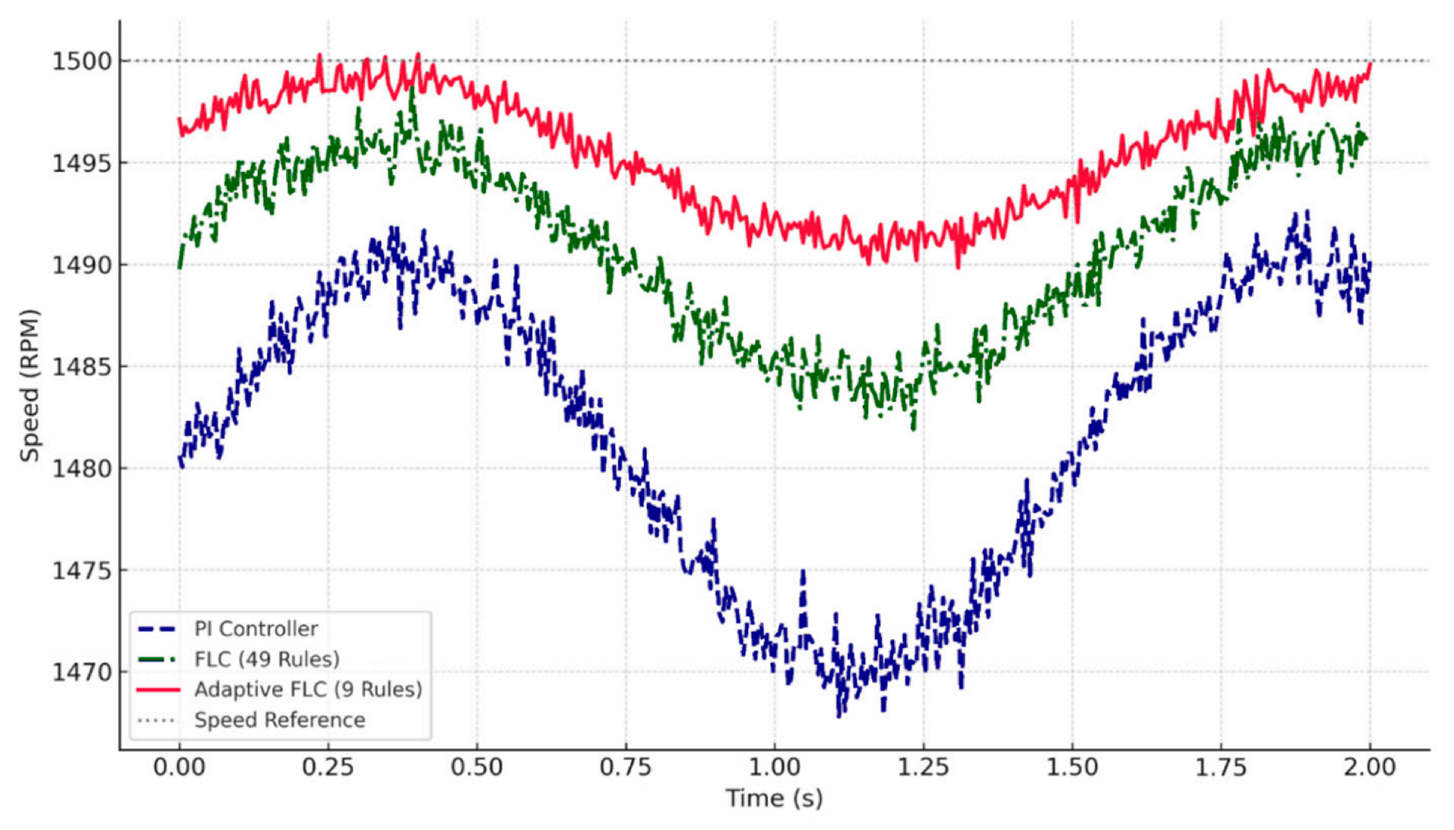
Speed response comparison under time-varying random load torque disturbance for the PI controller, classical 49-rule FLC, and proposed 9-rule adaptive FLC.

Figure 10 illustrates the rotor speed responses of the three control strategies under the random load torque disturbance. The PI controller exhibits pronounced deviations from the reference speed, accompanied by persistent oscillations throughout the operating interval. These fluctuations indicate limited disturbance rejection capability when subjected to unstructured and rapidly varying load conditions.

The classical 49-rule fuzzy logic controller demonstrates improved resilience compared to the PI controller. Speed deviations are reduced, and the oscillatory behavior is less severe. However, noticeable degradation in tracking performance is still observed during intervals of high torque fluctuation, suggesting that the fixed inference structure of the controller is not always sufficient to cope with continuously changing disturbance patterns.

In contrast, the proposed nine-rule adaptive fuzzy logic controller maintains close tracking of the reference speed with minimal oscillations across the entire test duration. The adaptive gain mechanism allows the controller to dynamically adjust the control effort in response to varying disturbance intensity, while the reduced rule base promotes smoother and more focused control action. This combination results in enhanced stability and improved rejection of non-deterministic load disturbances.

The quantitative performance metrics summarized in Table 9 further support these observations. The proposed controller achieves the lowest mean speed tracking error, the smallest standard deviation, and the minimum peak deviation among the evaluated strategies. In addition, it requires the least control effort, indicating improved energy efficiency and reduced actuation activity under stochastic loading conditions.

Overall, this test case confirms the ability of the proposed minimal-rule adaptive fuzzy logic controller to operate reliably under nonlinear, time-varying, and unpredictable load torque disturbances. The results demonstrate that combining a compact fuzzy structure with self-tuning gain adaptation provides an effective low-rule-count control strategy under the investigated uncertain-load conditions.

## 6. Discussion Summary

The conducted simulation study, supported by comprehensive quantitative benchmarks, demonstrates that the proposed nine-rule adaptive fuzzy logic controller achieves an effective balance between dynamic tracking performance, robustness, and computational efficiency. Across the evaluated operating scenarios, the controller consistently delivers fast transient response, high steady-state accuracy, and stable behavior under both deterministic and stochastic load conditions.

In terms of transient performance, the proposed controller exhibits the shortest rise time (0.15 s) and a minimal steady-state speed error of 4 RPM, while maintaining a well-damped response with negligible overshoot. These results closely match, and in several cases surpass, the performance of the classical 49-rule fuzzy logic controller, despite relying on a substantially reduced rule base.

A key factor contributing to this performance is the integrated adaptive gain mechanism, which dynamically modulates the control effort based on the instantaneous speed error. This feature enables aggressive corrective action during large disturbances and reference changes, while gradually attenuating the control gain as steady-state conditions are approached. As a result, the controller achieves rapid convergence without inducing excessive oscillations or unnecessary control activity.

The effectiveness of this adaptive strategy is particularly evident in the load disturbance rejection tests, including both step and time-varying random torque scenarios. In these cases, the proposed controller demonstrates superior disturbance rejection capability and improved stability compared to the benchmark controllers, while simultaneously reducing control effort and computational burden.

Overall, the discussion confirms that high-performance speed regulation does not necessarily require a large or complex fuzzy rule base. Instead, a carefully designed minimal-rule structure, when augmented with adaptive tuning mechanisms, can provide efficient control and effective rejection of the load-torque disturbances investigated in this study.

The robustness assessment in this study covers deterministic and stochastic load-torque disturbances and the digital non-idealities included in the fixed-step simulation environment. Specifically, the controller was evaluated under a load-torque step from 0.5 to 1.2 Nm and under a filtered pseudo-random torque disturbance limited to ±0.7 Nm. These tests demonstrate disturbance rejection capability over the investigated loading conditions. However, independent parameter sweeps of rotor resistance and total inertia, as well as a systematic variation of the sampling period, were not performed in the present study. Accordingly, the robustness conclusions are limited to the modeled load disturbances and the adopted sampling period of 100 μs; broader parametric robustness remains to be established through dedicated sensitivity analysis. The robustness assessment in this study is limited to the deterministic and stochastic load-torque disturbances and the fixed sampling period examined herein. Specifically, the controller was evaluated under a load-torque step from 0.5 to 1.2 Nm and a filtered pseudo-random torque disturbance limited to ±0.7 Nm. Independent parameter sweeps of rotor resistance, total inertia, and sampling period were not performed. Therefore, broader parametric-robustness conclusions require dedicated sensitivity analyses involving rotor-resistance variation, inertia mismatch, and sampling-period changes.

## 7. Discrete-Time Simulation Under Modeled Digital Non-Idealities

To evaluate the proposed controller under modeled digital non-idealities, an additional fixed-step discrete-time simulation was conducted incorporating measurement noise, signal quantization, and a one-sample computational delay. Its purpose was limited to assessing preservation of the simulated closed-loop response under these selected effects. Generated-code SIL, HIL, and target-processor validation remain outside the scope of the present study.

### 7.1. Discrete-Time Simulation Environment

Unlike the reference simulations presented earlier, the additional discrete-time simulation introduces selected digital non-idealities relevant to digitally implemented drive systems. First, the controller is executed with a fixed sampling period of $T_{s}=100\;\mu s$, reflecting the discrete nature of typical DSP-based control loops. Second, sensor imperfections are emulated by injecting Gaussian white noise with a standard deviation of approximately 1.5 RPM into the speed feedback signal. Finally, a one-sample delay ($z^{-1}$) is introduced as a modeled loop-latency effect.

The delay was applied without a predictor, phase-lead compensator, or explicit dead-time compensation. Accordingly, the reported response reflects the inherent tolerance of the closed loop to the imposed one-sample latency rather than the effect of an additional delay-compensation algorithm.

**Figure 11:**
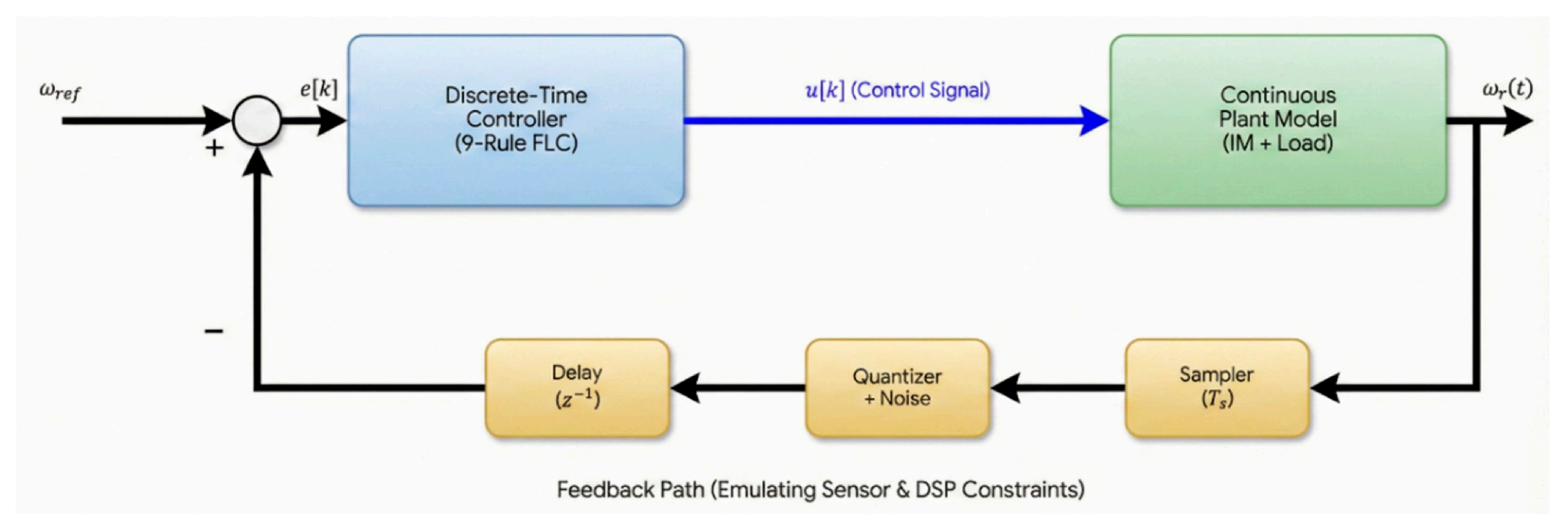
Discrete-time control-loop model, including measurement noise, signal quantization, and one-sample delay.

The overall discrete-time control architecture, including the sampler, quantization, and delay elements, is illustrated in Figure 11. This configuration provides a simulation platform for evaluating the controller response under the selected digital non-idealities.

### 7.2. Discrete-Time Performance Results

#### 7.2.1. Step Response Under Noise and Delay

**Figure 12:**
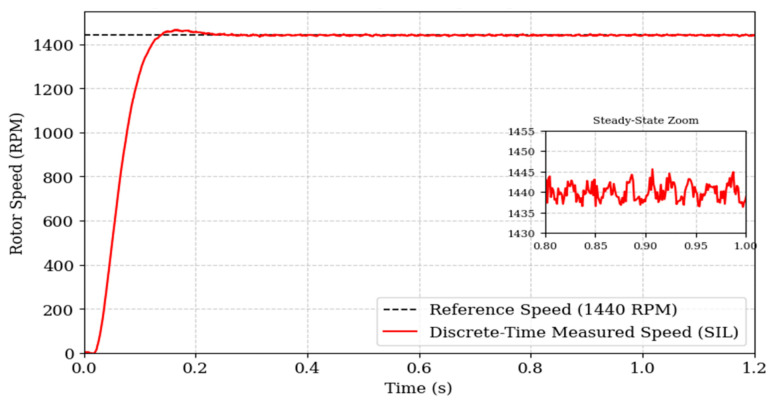
Discrete-time speed response.

Figure 12 illustrates the rotor speed response obtained from the fixed-step discrete-time simulation incorporating measurement noise, signal quantization, and one-sample delay. With the uncompensated one-sample delay, measurement noise, and signal quantization included in the simulation, the proposed controller retains a stable and well-damped response under the investigated conditions. This result indicates tolerance to the specific 100 μs delay represented in the model, but it does not establish a general delay margin or phase-margin guarantee. In steady-state operation, the speed fluctuations remain confined within a narrow band of approximately ±6 RPM, indicating strong noise tolerance.

These results show that the reduced nine-rule fuzzy structure preserves its simulated closed-loop performance under the adopted sampling, noise, quantization, and one-sample-delay conditions.

#### 7.2.2. Robustness to Load Disturbances in Discrete Time

**Figure 13:**
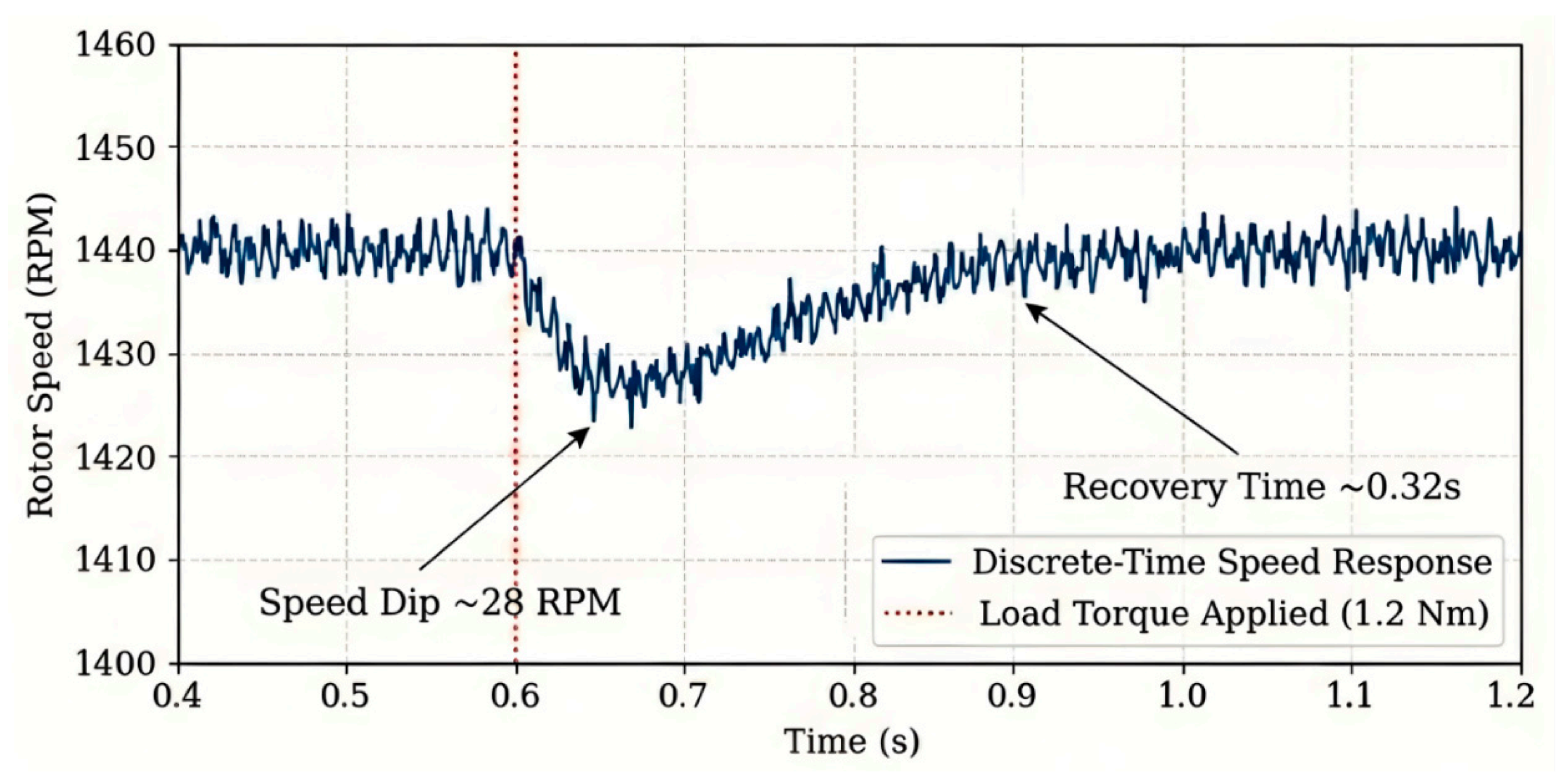
Discrete-time load disturbance.

The robustness test was repeated under the discrete-time simulation with the modeled digital non-idealities by applying a load torque of 1.2 Nm at t=0.6 s, as shown in Figure 13. Following the disturbance, the rotor speed experiences a temporary dip of approximately 28 RPM. The controller then restores the speed smoothly, with a recovery time of about 0.32 s.

Importantly, the adaptive gain mechanism remains fully effective under discrete-time operation, enabling rapid corrective action without inducing oscillations or instability. This confirms that the self-tuning behavior of the controller is not compromised by noisy or discretized feedback signals.

#### 7.2.3. Comparative Assessment with Ideal Simulation

Table 10 compares the reference simulation results with those obtained after introducing the modeled digital non-idealities. The observed differences are limited mainly to a modest increase in rise time and steady-state variation. The preservation of a stable and well-damped response indicates tolerance to the specific sampling, noise, quantization, and delay conditions represented in the simulation. These results do not constitute SIL, HIL, or processor-level validation.

Table 10 summarizes the effect of the modeled digital non-idealities on the simulated speed-tracking response. The rise time increases modestly from 0.15 s to 0.17 s, while the response remains well damped with negligible overshoot. The post-transient mean absolute tracking error increases from 4 RPM to approximately 6 RPM, primarily because measurement noise and signal quantization introduce residual fluctuations within the evaluation window. This quantity represents a finite-window tracking metric and should not be interpreted as the exact asymptotic steady-state offset. The results indicate that the closed-loop response remains stable under the specific sampling, quantization, noise, and one-sample-delay conditions investigated in the simulation.

## 8. Conclusions

This study presented a minimal-rule adaptive fuzzy logic controller for high-performance induction motor speed control, with particular emphasis on balancing dynamic performance and structural algorithmic simplicity. By reducing the fuzzy rule base from 49 to 9 rules and combining it with a bounded adaptive output gain mechanism, the proposed controller substantially decreases the maximum number of fuzzy-rule evaluations per control update while preserving strong tracking performance under the investigated reference and load-disturbance scenarios.

The principal contribution lies in separating computationally intensive parameter tuning from lightweight online adaptation. Particle Swarm Optimization is employed exclusively during the offline design stage to determine the nominal gain and normalized-error sensitivity coefficient. During each online control update, the controller requires only nine-rule fuzzy inference and a bounded scalar-gain update, without iterative optimization, rule reconstruction, or membership-function retuning.

The comparative simulation results show that the proposed controller outperforms the conventional PI controller and achieves performance comparable to, or better than, that of the classical 49-rule FLC in most evaluated tracking metrics. It provides a rise time of 0.15 s, a settling time of 0.26 s, a post-transient mean absolute tracking error of 4 RPM, and negligible overshoot. The classical 49-rule FLC retains a modest advantage in torque-ripple attenuation, achieving approximately 0.38 Nm compared with 0.44 Nm for the proposed controller. These results indicate that the bounded adaptive-gain mechanism compensates effectively for the reduced rule-base resolution over the investigated operating envelope.

The proposed strategy was also examined through an additional fixed-step discrete-time simulation incorporating measurement noise, 12-bit signal quantization, and a one-sample computational delay. Under these modeled digital non-idealities, the simulated closed-loop response remained stable and well damped, with only modest degradation relative to the reference simulation. However, no generated C/C++ code, simulated target environment, Hardware-in-the-Loop platform, or physical processor was used. Accordingly, this assessment should not be interpreted as generated-code SIL, HIL, or target-processor validation.

The computational comparison was therefore restricted to directly verifiable structural indicators, including rule-base size, the maximum number of fuzzy-rule evaluations per control update, and the bounded scalar-gain update. Relative to the classical 49-rule FLC, the proposed controller reduces the maximum number of fuzzy-rule evaluations from 49 to 9, corresponding to an 81.6% structural reduction. MATLAB Profiler measurements were excluded because host-computer timings do not represent execution time, worst-case latency, memory behavior, or processor utilization on a target microcontroller or DSP.

The robustness conclusions are limited to the deterministic and stochastic load-torque disturbances and the fixed sampling period investigated in this study. Independent sensitivity analyses involving rotor-resistance variation, inertia mismatch, and sampling-period changes were not performed and remain necessary before broader parametric-robustness claims can be established. In addition, the Lyapunov analysis provides a sufficient conditional stability criterion within the stated admissible sector rather than a numerically verified contraction margin.

Overall, the results demonstrate that strong simulation-level speed-control performance can be achieved using a substantially reduced fuzzy rule base when combined with bounded adaptive-gain tuning. The proposed controller therefore provides a favorable trade-off between dynamic performance and structural algorithmic complexity and should be regarded as a low-complexity candidate for future embedded evaluation rather than an embedded-ready solution. Generated-code SIL, HIL testing, target-processor timing and memory measurements, parameter-sensitivity studies, and experimental motor-drive validation constitute the principal directions for future work.

## Figures and Tables

**Table 1:** Parameters of the induction motor.

Parameter	Symbol	Value	Unit
Rated Power	P_rated_	1.5	kW
Rated Voltage	V_rated_	220	V
Rated Frequency	f	50	Hz
Rated Speed	ω_rated_	1440	RPM
Pole Pairs	p	2	-
Stator Resistance	R_s_	4.85	Ω
Rotor Resistance	R_r_	3.805	Ω
Stator Inductance	L_s_	0.274	H
Rotor Inductance	L_r_	0.274	H
Mutual Inductance	L_m_	0.258	H
Moment of Inertia	J	0.031	kg·m^2^
Friction Coefficient	B	0.00114	N·m·s/rad

**Table 2:** Scaling and normalized universes of discourse.

Variable	Physical Range	Normalized Range	Scaling Factor
Speed error, $e\left(k\right)$	$\left[-1440,1440\right]$ RPM	$\left[-1,1\right]$	$K_{e}=1/1440\;{RPM}^{-1}$
Error increment, $\Delta e\left(k\right)$	$\left[-1440,1440\right]$ RPM/sample	$\left[-1,1\right]$	$K_{\Delta e}=1/1440\;{RPM}^{-1}$
Fuzzy output, $u_{FLC}\left(k\right)$	Dimensionless	$\left[-1,1\right]$	Scaled by K(k) after defuzzification

**Table 3:** Membership-function parameters used for both inputs and the output.

Linguistic Set	Shape	Numerical Parameters
**Negative (N)**	Trapezoidal shoulder	$\left[-1,-1,-0.5,0\right]$
**Zero (Z)**	Triangular	$\left[-0.5,0,0.5\right]$
**Positive (P)**	Trapezoidal shoulder	$\left[0,0.5,1,1\right]$

**Table 4:** Complete 3 × 3 fuzzy rule table for *u*_FLC_ (*k*).

$e_{n}\left(k\right)$ $\Delta e_{n}\left(k\right)$	N	Z	P
N	N	N	Z
Z	N	Z	P
P	Z	P	P

**Table 5:** PSO-derived controller parameters and analytically derived adaptive-gain bounds.

Parameter	Description	Best-Performing Value
$k_{0}$	Base Output Gain	1.85
α	Dimensionless normalized-error sensitivity coefficient	0.12
W	PSO Inertia Weight	0.9 to 0.4
$c_{1},c_{2}$	Acceleration Coefficients	2.0
$K_{min}$	Minimum adaptive output gain	1.85
$K_{max}$	Maximum adaptive output gain	2.072

**Table 6:** Simulation configuration parameters.

Parameter	Value
Sampling Time (Ts)	100 μs
Solver Type	Fixed-step, Discrete
Computational Delay	1Ts (z^−1^)
Noise Model	Gaussian White Noise
Control Domain	Discrete-time (z-domain)
Digital Assessment Method	Fixed-step discrete-time simulation with modeled non-idealities
Speed-feedback noise	Gaussian, σ = 1.5 RPM, σ^2^ = 2.25 RPM^2^
Load disturbance	0.5 → 1.2 Nm at t = 0.6 s
Quantization	12 bit over [−1440, 1440] RPM; 0.703 RPM/LSB

**Table 7:** Quantitative performance comparison under step speed reference.

Controller Strategy	Rise Time (t_r_) [s]	Settling Time (t_s_) [s]	Post-Transient Mean Absolute Tracking Error (e _MAE,post_) [RPM]	Overshoot (Mp) [%]
Conventional PI	0.21	0.35	18.0	Negligible
Classical FLC (49 Rules)	0.18	0.28	8.0	Negligible
Proposed FLC (9 Rules)	0.15	0.26	4.0	Negligible

**Table 8:** Structural algorithmic complexity of the evaluated controllers.

Controller	Rule-Base Size	Fuzzy-Rule Evaluations Per Update	Online Adaptation	Interpretation
**Conventional PI**	Not applicable	0	No	Lowest structural complexity
**Classical FLC**	49	Up to 49	No	Highest fuzzy-inference complexity
**Proposed FLC**	9	Up to 9	Scalar gain update	Reduced fuzzy-inference complexity

**Table 9:** Performance under time-varying random torque disturbance.

Controller Strategy	Mean Speed Error (RPM)	Std. Deviation (RPM)	Max Deviation (RPM)	Control Effort (Joules)
Conventional PI	12.4	6.8	28.2	4.71
Classical FLC (49 Rules)	6.7	3.5	14.5	3.82
Proposed FLC (9 Rules)	4.2	2.1	9.8	2.94

**Table 10:** Effect of modeled digital non-idealities on the simulated speed-tracking response.

Performance Metric	Reference Simulation	Simulation with Modeled Digital Non-Idealities	Observed Effect
**Rise time, tr**	0.15 s	0.17 s	Slight increase associated with discrete sampling and the modeled one-sample delay
**Overshoot**	Negligible	Negligible	Well-damped response retained under the investigated conditions
**Post-transient mean** **absolute tracking error, e_MAE,post_**	4 RPM	Approximately 6 RPM	Increase associated primarily with measurement noise and signal quantization
**Closed-loop behavior under digital non-idealities**	Reference numerical conditions	Ts = 100 microseconds, 12-bit quantization, measurement noise, and one-sample delay	Stable simulated response under the adopted digital non-idealities

## Data Availability

The original contributions presented in this study are included in the article. Further inquiries can be directed to the corresponding authors.

## References

[1] Bose B.K. (1986). Power Electronics and AC Drives.

[2] Brdys M.A., Kulawski G.J. (1999). Dynamic neural controllers for induction motor. IEEE Trans. Neural Netw..

[3] Munoz-Garcia A., Lipo T.A., Novotny D.W. (1998). A new induction motor V/f control method capable of high-performance regulation at low speeds. IEEE Trans. Ind. Appl..

[4] Wang F., Zhang Z., Mei X., Rodríguez J., Kennel R. (2018). Advanced control strategies of induction machine: Field oriented control, direct torque control and model predictive control. Energies.

[5] Uddin M.N., Radwan T.S., Rahman M.A. (2002). Performances of fuzzy-logic-based indirect vector control for induction motor drive. IEEE Trans. Ind. Appl..

[6] Tarbosh Q.A., Aydogdu O., Farah N., Talib H.N., Salh A., Cankaya N., Omar F.A., Durdu A. (2020). Review and investigation of simplified rules fuzzy logic speed controller of high performance induction motor drives. IEEE Access.

[7] Tang H.H., Ahmad N.S. (2024). Fuzzy logic approach for controlling uncertain and nonlinear systems: A comprehensive review of applications and advances. Syst. Sci. Control Eng..

[8] Lamamra K., Batat F., Mokhtari F. (2020). A new technique with improved control quality of nonlinear systems using an optimized fuzzy logic controller. Expert Syst. Appl..

[9] Arulmozhiyaly R., Baskaran K. (2010). Implementation of a fuzzy PI controller for speed control of induction motors using FPGA. J. Power Electron..

[10] Elhawat M., Altınkaya H. (2025). Frequency Regulation of Stand-Alone Synchronous Generator via Induction Motor Speed Control Using a PSO-Fuzzy PID Controller. Appl. Sci..

[11] Albertos P., Sala A., Olivares M. (1998). Fuzzy logic controllers. Advantages and drawbacks. VIII International Congress of Automatic Control.

[12] Li Z., Dewantoro G., Xiao T., Swain A. (2025). A Comparative Analysis of Fuzzy Logic Control and Model Predictive Control in Photovoltaic Maximum Power Point Tracking. Electronics.

[13] Duch W., Adamczak R., Grabczewski K. (2001). A new methodology of extraction, optimization and application of crisp and fuzzy logical rules. IEEE Trans. Neural Netw..

[14] Zellouma D., Bekakra Y., Benbouhenni H. (2023). Field-oriented control based on parallel proportional–integral controllers of induction motor drive. Energy Rep..

[15] Alnaib I., Alsammak A. (2025). Optimization of fractional PI controller parameters for enhanced induction motor speed control via indirect field-oriented control. Electr. Eng. Electromech..

[16] Mamdani E.H., Assilian S. (1975). An experiment in linguistic synthesis with a fuzzy logic controller. Int. J. Man-Mach. Stud..

[17] Zadeh L.A. (1965). Fuzzy sets. Inf. Control.

[18] Zaky M.S., Metwaly M.K. (2016). A performance investigation of a four-switch three-phase inverter-fed IM drives at low speeds using fuzzy logic and PI controllers. IEEE Trans. Power Electron..

[19] Hemeyine A.V., Abbou A., Bakouri A., Mokhlis M., El Moustapha S.M.O.M. (2021). A robust interval Type-2 fuzzy logic controller for variable speed wind turbines based on a doubly fed induction generator. Inventions.

[20] Talib M., Ibrahim Z., Rahim N.A., Zulhani R., Nordin N., Farah N., Razali A. (2020). An improved simplified rules Fuzzy Logic Speed Controller method applied for induction motor drive. ISA Trans..

[21] Apribowo C.H.B., Maghfiroh H. (2021). Fuzzy logic controller and its application in brushless DC motor (BLDC) in electric vehicle-a review. J. Electr. Electron. Inf. Commun. Technol..

[22] Vargas O.S., Aldaco S.E.D.L., Alquicira J.A., Vela-Valdés L.G., Núñez A.R.L. (2023). Adaptive network-based fuzzy inference system (ANFIS) applied to inverters: A survey. IEEE Trans. Power Electron..

[23] Nguyen C.T., Nguy B.-H., Trovão J.P.F., Ta M.C. (2023). Torque distribution optimization for a dual-motor electric vehicle using adaptive network-based fuzzy inference system. IEEE Trans. Energy Convers..

[24] Kumar A.S., Naveen S., Vijayakumar R., Suresh V., Asary A.R., Madhu S., Palani K. (2023). An intelligent fuzzy-particle swarm optimization supervisory-based control of robot manipulator for industrial welding applications. Sci. Rep..

[25] Ibrahim O., Aziz M.J.A., Ayop R., Dahiru A.T., Low W.Y., Sulaiman M.H., Amosa T.I. (2024). Fuzzy logic-based particle swarm optimization for integrated energy management system considering battery storage degradation. Results Eng..

[26] Maroua B., Laid Z., Benbouhenni H., Elbarbary Z., Colak I., Alammer M.M. (2025). Genetic algorithm type 2 fuzzy logic controller of microgrid system with a fractional-order technique. Sci. Rep..

[27] Rodríguez-Abreo O., Rodríguez-Reséndiz J., García-Cerezo A., García-Martínez J.R. (2024). Fuzzy logic controller for UAV with gains optimized via genetic algorithm. Heliyon.

[28] Sastry K.K.N., Behera L., Nagrath I.J. (1999). Differential Evolution Based Fuzzy Logic Controller for Nonlinear Process Control. Fundam. Inform..

[29] Ochoa P., Castillo O., Soria J. (2020). Optimization of fuzzy controller design using a Differential Evolution algorithm with dynamic parameter adaptation based on Type-1 and Interval Type-2 fuzzy systems. Soft Comput..

[30] Kamthan S., Singh H. (2022). Hierarchical fuzzy logic systems. J. Inst. Eng. Ser. B.

[31] Mendel J.M. (2024). Explainable Uncertain Rule-Based Fuzzy Systems.

[32] Suetake M., da Silva I.N., Goedtel A. (2010). Embedded DSP-based compact fuzzy system and its application for induction-motor V/f speed control. IEEE Trans. Ind. Electron..

[33] Woźniak M., Zielonka A., Sikora A. (2022). Driving support by type-2 fuzzy logic control model. Expert Syst. Appl..

[34] Puška A., Nedeljković M., Zolfani S.H., Pamučar D. (2021). Application of interval fuzzy logic in selecting a sustainable supplier on the example of agricultural production. Symmetry.

[35] Schwenzer M., Ay M., Bergs T., Abel D. (2021). Review on model predictive control: An engineering perspective. Int. J. Adv. Manuf. Technol..

[36] Omar F.A. Sliding Mode Control for DC-DC Converters: Evaluation and Analysis. Proceedings of the 3rd International Conference on Scientific and Academic Research.

[37] Menaem A.A., Elgamal M., Abdel-Aty A.-H., Mahmoud E.E., Chen Z., Hassan M.A. (2020). A proposed ANN-based acceleration control scheme for soft starting induction motor. IEEE Access.

[38] Talpur N., Abdulkadir S.J., Alhussian H., Hasan M.H., Aziz N., Bamhdi A. (2022). A comprehensive review of deep neuro-fuzzy system architectures and their optimization methods. Neural Comput. Appl..

[39] Anwer A.M.O., Omar F.A., Kulaksiz A.A. (2020). Design of a fuzzy logic-based MPPT controller for a PV system employing sensorless control of MRAS-based PMSM. Int. J. Control Autom. Syst..

[40] Maia R., Silva M., Araújo R., Nunes U. (2015). Electrical vehicle modeling: A fuzzy logic model for regenerative braking. Expert Syst. Appl..

[41] Aliworom C., Uzoechi L., Olubiwe M. (2018). Design of fuzzy logic tracking controller for industrial conveyor system. Int. J. Eng. Trends Technol..

[42] Khooban M.H., Gheisarnejad M. (2020). A novel deep reinforcement learning controller based type-II fuzzy system: Frequency regulation in microgrids. IEEE Trans. Emerg. Top. Comput. Intell..

[43] Khalid K., Woungang I., Dhurandher S.K., Singh J. (2021). Reinforcement learning-based fuzzy geocast routing protocol for opportunistic networks. Internet Things.

[44] Vu N.T.-T., Nguyen H.D., Nguyen A.T. (2022). Reinforcement learning-based adaptive optimal fuzzy MPPT control for variable speed wind turbine. IEEE Access.

[45] Kecskés I., Székács L., Odry P. Lookup table based fuzzy controller implementation in low-power microcontrollers of hexapod robot Szabad (ka)-II. Proceedings of the 3rd International Conference & Workshop Mechatronics in Practice and Education–MECHEDU.

[46] Benbouali A., Chabni F., Taleb R., Mansour N. (2021). Flight parameters improvement for an unmanned aerial vehicle using a lookup table based fuzzy PID controller. Indones. J. Electr. Eng. Comput. Sci..

[47] Kojabadi H.M. (2005). Simulation and experimental studies of model reference adaptive system for sensorless induction motor drive. Simul. Model. Pract. Theory.

[48] Mezouar A., Fellah M.K., Hadjeri S. (2008). Adaptive sliding-mode-observer for sensorless induction motor drive using two-time-scale approach. Simul. Model. Pract. Theory.

[49] Obeid H., Laghrouche S., Fridman L., Chitour Y., Harmouche M. (2019). Barrier function-based variable gain super-twisting controller. arXiv.

[50] Ibraheem I.K., Humaidi A.J. (2019). On the design of a novel finite-time nonlinear extended state observer for a class of nonlinear systems with mismatch disturbances and uncertainties. arXiv.

[51] Fonseca A.G., Duarte R.A., Albuquerque K.S., Pinto T.V.B., Euzébio T.A.M. (2026). Mitigating overload conditions in vibrating screens through fuzzy-controlled feed rate regulation. Miner. Eng..

